# Selective autophagy maintains centrosome integrity and accurate mitosis by turnover of centriolar satellites

**DOI:** 10.1038/s41467-019-12094-9

**Published:** 2019-09-13

**Authors:** Søs Grønbæk Holdgaard, Valentina Cianfanelli, Emanuela Pupo, Matteo Lambrughi, Michal Lubas, Julie C. Nielsen, Susana Eibes, Emiliano Maiani, Lea M. Harder, Nicole Wesch, Mads Møller Foged, Kenji Maeda, Francesca Nazio, Laura R. de la Ballina, Volker Dötsch, Andreas Brech, Lisa B. Frankel, Marja Jäättelä, Franco Locatelli, Marin Barisic, Jens S. Andersen, Simon Bekker-Jensen, Anders H. Lund, Vladimir V. Rogov, Elena Papaleo, Letizia Lanzetti, Daniela De Zio, Francesco Cecconi

**Affiliations:** 1 Cell Stress and Survival Unit, Center for Autophagy, Recycling and Disease (CARD), Danish Cancer Society Research Center, Copenhagen, 2100 Denmark; 2 Department of Oncology, University of Torino Medical School, Turin, 10100 Italy; 3 Candiolo Cancer Institute, FPO - IRCCS, Candiolo, 10060 Italy; 4 Computational Biology Laboratory, Center for Autophagy, Recycling and Disease (CARD), Danish Cancer Society Research Center, Copenhagen, 2100 Denmark; 5 Biotech Research & Innovation Centre, University of Copenhagen, Copenhagen, 2200 Denmark; 6 Center for Healthy Aging, University of Copenhagen, Copenhagen, 2200 Denmark; 7 Cell Division Laboratory, Danish Cancer Society Research Center, Copenhagen, 2100 Denmark; 8 Department of Cellular and Molecular Medicine, University of Copenhagen, Copenhagen, 2200 Denmark; 9 Department of Biochemistry and Molecular Biology, University of Southern Denmark, Odense, 5230 Denmark; 10 Institute of Biophysical Chemistry and Center for Biomolecular Magnetic Resonance, Goethe University Frankfurt, 60438 Frankfurt, Germany; 11 Cell Death and Metabolism Unit, Center for Autophagy, Recycling and Disease (CARD), Danish Cancer Society Research Center, Copenhagen, 2100 Denmark; 12 Department of Pediatric Hemato-Oncology and Cell and Gene therapy, IRCCS Bambino Gesù Children’s Hospital, Rome, 00143 Italy; 13 Department of Molecular Medicine, Institute of Basic Medical Sciences and Centre for Cancer Cell Reprogramming, Institute of Clinical Medicine, Faculty of Medicine, University of Oslo, 0317 Oslo, Norway; 14 Department of Molecular Cell Biology, Institute for Cancer Research, Oslo University Hospital, 0379 Oslo, Norway; 15 Centre for Cancer Cell Reprogramming, Institute of Clinical Medicine, Faculty of Medicine, University of Oslo, 0379 Oslo, Norway; 16 RNA and Autophagy group, Danish Cancer Society Research Center, Copenhagen, 2100 Denmark; 17 Department of Cellular and Molecular Medicine, Faculty of Health Sciences, University of Copenhagen, Copenhagen, 2200 Denmark; 18 Department of Gynecology/Obstetrics and Pediatrics, Sapienza University of Rome, Rome, 00185 Italy; 19 Translational Disease Systems Biology, Faculty of Health and Medical Sciences, Novo Nordisk Foundation Center for Protein Research University of Copenhagen, Copenhagen, 2100 Denmark; 20 Department of Biology, University of Tor Vergata, Rome, 00133 Italy

**Keywords:** Macroautophagy, Mitosis

## Abstract

The centrosome is the master orchestrator of mitotic spindle formation and chromosome segregation in animal cells. Centrosome abnormalities are frequently observed in cancer, but little is known of their origin and about pathways affecting centrosome homeostasis. Here we show that autophagy preserves centrosome organization and stability through selective turnover of centriolar satellite components, a process we termed doryphagy. Autophagy targets the satellite organizer PCM1 by interacting with GABARAPs via a C-terminal LIR motif. Accordingly, autophagy deficiency results in accumulation of large abnormal centriolar satellites and a resultant dysregulation of centrosome composition. These alterations have critical impact on centrosome stability and lead to mitotic centrosome fragmentation and unbalanced chromosome segregation. Our findings identify doryphagy as an important centrosome-regulating pathway and bring mechanistic insights to the link between autophagy dysfunction and chromosomal instability. In addition, we highlight the vital role of centriolar satellites in maintaining centrosome integrity.

## Abstract (web summary)

Centrosomes drive mitotic spindle formation and chromosome segregation. Here, the authors show that centrosome stability is regulated by selective autophagic degradation of centriolar satellite components in a process they term doryphagy, connecting autophagy and chromosomal integrity.

## Introduction

Macroautophagy (hereafter referred to as autophagy) is a highly conserved catabolic pathway, in which cytosolic material is delivered to the lysosome for degradation. This is achieved by sequestration of the cytosolic substrates in double-membrane vesicles, termed autophagosomes, that then fuse with lysosomes, whereby the engulfed cargo is degraded by lysosomal enzymes and subsequently released to the cytosol for recycling^1^.

While autophagy was initially considered a non-selective process, it has now been established that the pathway displays selectivity in many contexts. Indeed, autophagy can specifically recruit larger structures, such as organelles and protein complexes, for autophagic degradation^2^. Cargo recognition is achieved through substrate binding to autophagy-related gene (ATG) 8 family proteins (the LC3 and GABARAP sub-families in mammals) present on the internal side of the autophagosome, physically linking the cargo to the autophagic vesicle. ATG8 binding can be mediated by binding to intrinsic LC3-interacting region (LIR) motifs in the substrate, or occur through specialized autophagy receptors, that simultaneously interact with the substrate and ATG8 variants on autophagosomes^2^. Identification of novel selective autophagy pathways is pivotal to dissect the multitude of cellular processes that are regulated by autophagy, and their relevance for human autophagy-linked pathologies^3,4^.

The centrosome is a small perinuclear organelle that functions as the major microtubule-organizing center (MTOC) of animal cells^5^. In its center, are two cylindrical structures, termed the centrioles, that are surrounded by a large electron-dense cloud of accessory proteins, collectively referred to as the pericentriolar material (PCM)^5,6^. The PCM contains hundreds of proteins organized in concentric toroids^7,8^, that orchestrate most aspects of centrosome functionality. In addition, associated with the microtubules emanating from the centrosome are the centriolar satellites (CS), small granular structures containing numerous centrosomal proteins. The CS are not yet understood in detail, but are known to play a vital role in the regulation of centrosome assembly, through delivery of components to the centrosome by dynein-mediated trafficking^9^. Their master organizer is a large self-associating protein, pericentriolar material 1 (PCM1), that is required for CS assembly, and accordingly, for the centrosomal recruitment of several key centrosome organizers and regulators, such as Pericentrin and centrin^9–11^.

The centrosome controls a plethora of cellular processes through its microtubule-nucleating capacity, including the nucleation and organization of the mitotic spindle, that is responsible for the equal distribution of the duplicated genome to the two daughter cells during mitosis^5,6^. This makes cell division particularly vulnerable to centrosome dysfunction. Accordingly, centrosome defects have been inextricably linked to chromosomal instability and are observed with a high frequency in human cancers^5,6^. It is, however, unclear how these abnormalities arise^6^. For this reason, identification of pathways that control centrosome homeostasis is fundamental for understanding the mechanisms of pathological centrosome dysfunction.

Here, we show a central role for autophagy in the regulation of centrosome stability. We find that depletion of key autophagy proteins results in mitotic centrosome fragmentation and chromosome segregation defects. Centrosome instability and fragmentation occur as a consequence of global disorganization of centrosome composition, due to failure of controlled turnover of CS by autophagy, leading to accumulation of large abnormal satellites. Autophagic CS degradation is achieved by targeting CS components through LIR-mediated interaction of GABARAPs with PCM1. Thus, we find that autophagy promotes selective CS turnover (doryphagy) to ensure CS functionality and the preservation of centrosome integrity.

## Results

### Impaired autophagy causes mitotic centrosome fragmentation

**Fig. 1:**
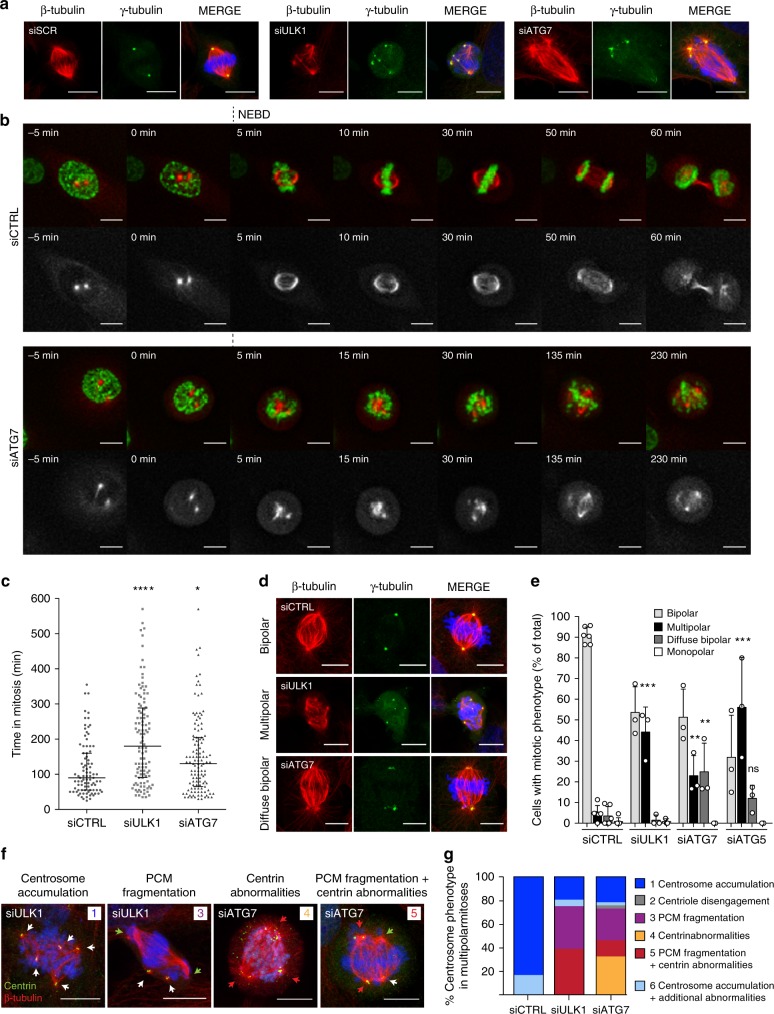
Depletion of key autophagy factors results in mitotic centrosome fragmentation. **a** Abnormal mitoses in U2OS cells treated with control, ULK1 or ATG7 siRNA stained for γ-tubulin, β-tubulin, and Hoechst33342. **b** Time-lapse imaging of stable U2OS mRFP-α-tubulin H2B-GFP cells treated with control or ATG7 siRNA. Images were acquired every 5 min for 10 h. Time after NEBD is indicated. **c** Quantification of experiments represented in (**b**) and Supplementary Fig. 1B. Time in mitosis was measured from NEBD to anaphase or until the end of the experiment. Results are pooled from three independent experiments. siSCR, *n* = 89; siULK1, *n* = 108; siATG7, *n* = 119. Bars represent medians and interquartile range. *P ≤ 0.05, ****P ≤ 0.0001. Two-tailed Mann–Whitney test. **d** Examples of phenotype categories; bipolar, multipolar and diffuse bipolar spindles. U2OS cells are stained for γ-tubulin, β-tubulin, and Hoechst33342. **e** Quantification of phenotype distribution in (**d**). Columns represent the mean ± SD, *n* = 3 of ≥20 cells, ns *P* > 0.05, **P ≤ 0.01, ***P ≤ 0.001. Unpaired Student’s *t*-test, two-tailed. **f** Multipolar mitoses in U2OS cells depleted of ULK1 or ATG7 analyzed by β-tubulin, centrin, and Hoechst33342 staining. Numbers refer to phenotype categories in (**g**). White arrow, spindle pole with two centrioles; green arrow, spindle pole without centrin (PCM fragmentation); red arrow, spindle pole with abnormal centrin **g**. Quantification of (**f**). Columns represent phenotype distribution of 3 pooled experiments. siSCR, *n* = 7; ULK1, *n* = 36; ATG7 *n* = 37. Scale bars, 10 µm. Source data are provided as a Source Data file

During our studies on the upstream regulation of autophagy, we noted the occurrence of abnormal mitoses when depleting key autophagy regulators. To further characterize these abnormalities, we transiently depleted the upstream autophagy regulator unc-51-like autophagy-activating kinase 1 (ULK1) or the more downstream factor ATG7 in U2OS cells (selected based on their good imaging properties), and analyzed the mitotic cells using γ-tubulin and β-tubulin as markers of centrosomes and microtubules, respectively. Remarkably, depletion of either autophagy protein resulted in the formation of aberrant mitoses with unstructured mitotic spindles and diffuse centrosomes (Fig. 1a). Efficient depletion of autophagy regulators and decreased autophagy flux was confirmed by immunoblotting (Supplementary Fig. 1a).

To explore the origin of the abnormal mitoses, ULK1 or ATG7 were depleted in U2OS cells stably expressing mRFP-α-tubulin and histone 2B-GFP (H2B-GFP), and the silenced cells were analyzed by live cell imaging. At the onset of mitosis, prior to nuclear-envelope break down (NEBD), ULK1- and ATG7-depleted cells contained two well-defined spindle poles, similar to control cells. However, following NEBD one or both spindle poles rapidly fragmented, often resulting in the formation of alternative spindle poles and multipolar mitoses (Fig. 1b, Supplementary Fig. 1B, Supplementary Movies 1A, B; 2A, B; 3A, B). These results strongly argue that the observed abnormal mitoses are generated by mitotic centrosome fragmentation. Spindle abnormalities were accompanied by a severe delay in anaphase onset in both ULK1- and ATG7-depleted cells (Fig. 1c). An analysis of prophase centrosomes confirmed that the centrosomes were intact prior to cell division in all conditions (Supplementary Fig. 1C).

To quantify the defective mitotic centrosomes in fixed cells, randomly selected mitoses were analyzed, following depletion of ULK1, ATG7 or ATG5, included as an additional control for autophagy specificity (see Supplementary Fig. 1 A). While control cells showed a low baseline of abnormalities, depletion of either autophagy regulator resulted in a high frequency of mitotic centrosome aberrations that could be divided in two categories: mitotic cells displaying multipolar spindle formation with >2 γ-tubulin foci or mitotic cells exhibiting diffuse bipolar spindles with γ-tubulin scattered over two poorly structured spindle poles (Fig. 1d, e). Pharmacological inhibition of ULK1 resulted in a gradual increase in mitotic abnormalities that resembled ULK1 depletion (Supplementary Fig. 1D, E). Furthermore, re-introduction of siRNA-resistant ATG7 markedly decreased the mitotic abnormalities resulting from ATG7-depletion (Supplementary Fig. 1F, G), confirming the specificity of the phenotype.

Autophagy deficiency has previously been linked to centrosome accumulation^12,13^. To confirm that the observed multipolar mitoses result from centrosome fragmentation rather than centrosome accumulation, a detailed analysis focusing specifically on the multipolar mitoses was performed. To this end, we examined the distribution of the centriole marker centrin^14^ at each spindle pole in the multipolar mitoses^15^. In ULK1- or ATG7-depleted cells multipolarity resulted from PCM fragmentation in ∼36% and ∼27% of cases, respectively (Fig. 1f, g), defined as mitotic cells presenting additional spindle poles devoid of centrioles^15^. Centriole disengagement, the splitting of coupled centrioles generating supernumerary poles with single centrioles^15^, was negligible (Fig. 1f, g). Approximately 20% of multipolar mitoses could be attributed to centrosome amplification (Fig. 1f, g). However, as the rare multipolar mitoses in the control U2OS culture were primarily the result of centrosome accumulation, we hypothesize that a minor level of centrosome accumulation is an intrinsic characteristic of the cell line. Indeed, interphase cells did not show augmented centrosome accumulation upon depletion of autophagy proteins (Supplementary Fig. 1H, I). Interestingly, a high number of multipolar mitoses contained large abnormal centrin structures and could not be distributed to the above categories, ∼39% for ULK1 and ∼45% for ATG7-depletion (Fig. 1f, g). To determine if the abnormal centrin foci constituted additional centrioles, CPAP, a centriole-associated protein involved in the regulation of centriole biogenesis^16^ was used as an alternative centriole marker. Indeed, CPAP did not co-localize with the superfluous centrin foci (Supplementary Fig. 2A), demonstrating that they do not represent centrioles. Thus, depletion of key autophagy regulators, results in mitotic centrosome fragmentation characterized by PCM fragmentation and abnormal centrin distribution.

### Autophagy affects DNA segregation and postmitotic cell death

**Fig. 2:**
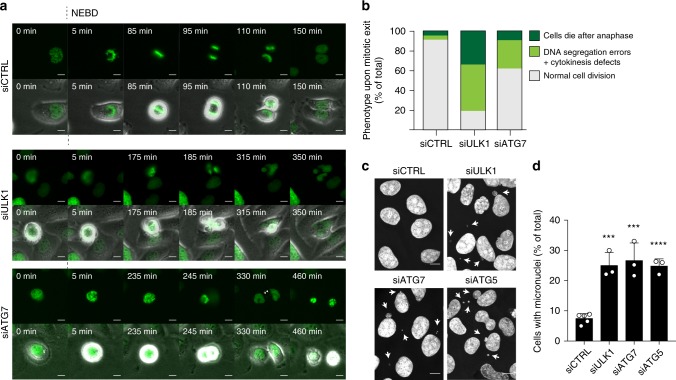
Autophagy deficiency leads to chromosome segregation defects and postmitotic cell death. **a** Time-lapse imaging experiments of stable U2OS mRFP-α-tubulin H2B-GFP cells transfected with control, ULK1 or ATG7 siRNAs. GFP and DIC channels were recorded. Time-points after NEBD are indicated. **b** Quantification of phenotype distribution shown in (**a**). SCR, *n* = 169; ULK1, *n* = 47; ATG7 *n* = 179. **c** Representative immunofluorescence images of Hoechst33342-stained U2OS cells treated with control, ULK1, ATG7 or ATG5 siRNA for micronuclei quantification. Arrows indicate micronuclei. **d** Quantification of experiments shown in (**c**). Columns represent the mean ± SD, *n* = 3 ≥ 100 cells, **P ≤ 0.01, ***P ≤ 0.001. Unpaired Student’s *t*-test, two-tailed. Scale bars, 10 µm. Source data are provided as a Source Data file

To investigate the effect of autophagy deficiency on mitotic exit and cell survival, combined acquisition of H2B-GFP and differential interference contrast (DIC) microscopy was employed in order to limit phototoxicity, allowing for longer image recording. In contrast to control cells, a substantial fraction of ULK1- or ATG7-depleted cells exhibited chromosome segregation defects. For ∼44% and ∼26% of ULK1 and ATG7-depleted cells, respectively, chromosome segregation occurred in a highly erroneous manner with occasional delayed cytokinesis, but the cells, nonetheless, survived the abnormal cell division (Fig. 2a, b, Supplementary Movies 4A, B; 5A, B). Conversely, in ∼34% (for ULK1 depletion) and ∼9% (for ATG7-depletion) of cases, one or both daughter cells died shortly after cell division (Fig. 2a, b, Supplementary Movie 6A, B). While the fidelity of chromosome segregation could not always be accurately determined for the latter events, most cells exhibited clear chromosome segregation defects prior to cell death (Fig. 2a), suggesting that, in these cases, the abnormalities were incompatible with cell survival.

The fidelity of DNA segregation was further evaluated by quantification of the cell fraction containing micronuclei, as micronuclei can be used as an indication of failure to incorporate all chromosomes or chromosome fragments in the reforming nuclei during cell division^17^. Depletion of ULK1, ATG7 or ATG5 all resulted in a high level of micronuclei formation (Fig. 2c, d), confirming that autophagy deficiency causes chromosome segregation defects.

### Autophagy deficiency results in accumulation of abnormal CS

**Fig. 3:**
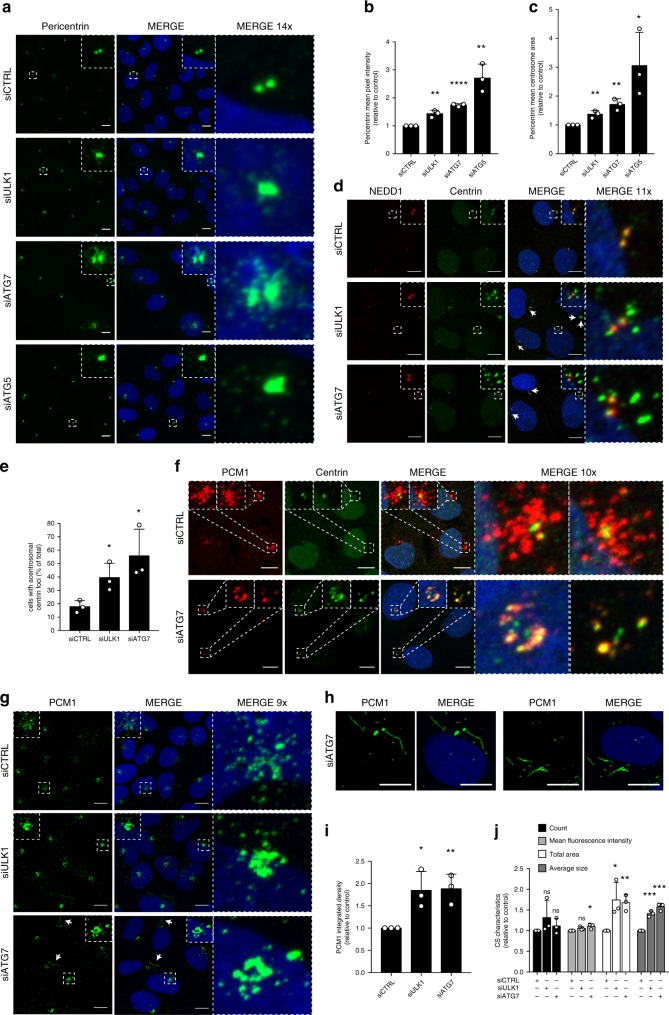
Interphase centrosomes display compositional changes and accumulation of abnormal CS upon autophagy deficiency. **a** U2OS cells transfected with control, ULK1, ATG7 or ATG5 siRNAs, stained for Pericentrin and Hoechst33342. **b**, **c** Quantification of (**a**). Columns represent mean fluorescence intensity (**b**) or mean centrosome area (**c**) ±SD, *n* = 3 of >60 cells. *P ≤ 0.05, **P ≤ 0.01, ****P ≤ 0.0001. Unpaired Student’s *t*-test, two-tailed. **d** Centrin distribution related to NEDD1-stained centrosomes in U2OS cells treated with control, ULK1 or ATG7 siRNAs. Arrows indicate examples of extra-centrosomal centrin foci. **e** Quantification of (**d**). Columns represent the mean frequency of acentrosomal centrin foci ±SD, *n* = 3 of > 100 cells. *P ≤ 0.05. Unpaired Student’s *t*-test, two-tailed. **f** Colocalization between centrin and PCM1 in U2OS cells transfected with control or ATG7 siRNAs. **g** PCM1 distribution in U2OS cells treated with control, ULK1 or ATG7 siRNAs. Arrows indicate string-like PCM1. **h** String-like distribution of PCM1 in ATG7-depleted cells. **i** Quantification of integrated density of experiments represented in (**g**). Columns represent the mean ± SD, *n* = 3 of >50 cells. *P ≤ 0.05, **P ≤ 0.01. Unpaired Student’s *t*-test, two-tailed. **j** Quantification of CS characteristics of experiments represented in (**g**). Columns represent the mean ± SD, *n* = 3 of >50 cells. ns *P* > 0.05, *P ≤ 0.05, **P ≤ 0.01, ***P ≤ 0.001. Unpaired Student’s *t*-test, two-tailed. Scale bars, 10 µm. Source data are provided as a Source Data file

Mitotic centrosome fragmentation has previously been reported as a consequence of centrosome compositional changes that compromise their stability and thus, their ability to withstand the traction forces exerted on them during bipolar spindle formation^18,19^. To understand if autophagy deficiency resulted in compositional alterations in the centrosomes, we performed a detailed analysis of interphase centrosomes, focusing on commonly used markers associated with distinct centrosome domains. To this end, we chose Pericentrin, a large structural protein involved in PCM recruitment and organization^7,20^, PCM-resident members of the microtubule-nucleating γ-TURC complex, NEDD1, and γ-tubulin^21^, centrin, as a marker of the centrioles and CEP63, a centrosome duplication factor^22^, previously linked to autophagy^12^. Strikingly, upon depletion of autophagy regulators, changes involving all centrosome markers were observed. This included a strong accumulation of Pericentrin at interphase centrosomes and scattered in their near vicinity (Fig. 3a–c), and a slight increase in the centrosomal level of NEDD1 and γ-tubulin (Supplementary Fig. 2B–D). CEP63 accumulated in a diffuse pattern in the immediate vicinity of the centrosome (Supplementary Fig. 2E) and centrin displayed a clear redistribution to aggregate-like foci surrounding the centrosome (Fig. 3d, e), resembling the structures observed in mitotic cells (see Fig. 1f). These centrin foci were also negative for the centriole marker CPAP (Supplementary Fig. 2F), suggesting that they do not represent centrioles.

To evaluate if the altered centrosome composition affected the microtubule nucleation capacity of the centrosomes, a microtubule regrowth assay was performed. Microtubule nucleation following cold-induced depolymerization was comparable in control and autophagy-deficient cells, as evaluated by the intensity and frequency of microtubule aster formation (Supplementary Fig. 2G–I), consistent with the modest changes observed in γ-TURC components (see Supplementary Fig. 2B–D). Furthermore, no changes in the overall microtubule organization was observed following longer intervals of regrowth (Supplementary Fig. 2G), suggesting that also microtubule anchoring was functional^23^.

As the centrosome is highly dynamic and undergoes dramatic changes during cell cycle^6^, the cell cycle profile was evaluated following depletion of autophagy regulators. No evident changes were observed (Supplementary Fig. 3A); thus, the composition of the centrosomes was not altered as a result of cellular redistribution to certain cell cycle phases.

Apart from altered centrosome organization, spindle pole fragmentation has also been linked to dysfunction of motor proteins required for bipolar spindle formation^24,25^. To determine if the autophagy-deficient cells displayed unbalanced motor protein levels, selected motors, the kinesin EG5 and selected components of the dynein-dynactin complex; dynein-intermediate-chain 1/2 (DIC1/2) and p150Glued, were evaluated. No changes in the protein level of either motor was observed (Supplementary Fig. 3B). Furthermore, the absence of monopolar spindle formation (see Fig. 1e), indicates that EG5 function was not impaired^26^. In addition, the microtubule-focusing factor NuMA that is recruited to the mitotic spindle poles by dynein-mediated transport following NEBD^27^, was still recruited to the aberrant spindle poles in autophagy-deficient cells (Supplementary Fig. 3C), suggesting that dynein-mediated transport was functional.

We then reasoned that the global and heterogeneous nature of the interphase centrosome changes could suggest dysregulation of upstream mechanisms, facilitating a general effect on centrosome organization. Thus, our interphase analyses prompted us to look closer at the CS. Interestingly, the acentrosomal centrin foci clearly co-localized with PCM1 (Fig. 3f), revealing that they represent accumulation of centrin at the CS. The CS are reported to undergo dispersal during mitosis^11^, leaving a low number of residual dispersed CS^28^. Of note, the mitotic supernumerary centrin foci (see Fig. 1f) co-localized with residual mitotic CS (Supplementary Fig. 3D), indicating that the superfluous interphase and mitotic centrin foci both represent abnormal CS relocation. The observed acentrosomal CEP63 and Pericentrin also partially co-localized with PCM1 (Supplementary Figs. 2E, 3E), suggesting that the global centrosome changes were linked to the CS. Strikingly, the satellites displayed a highly altered organization into larger abnormal structures and string-like formations in autophagy-deficient cells, with the latter more evident in ATG7-deficient cells (Fig. 3g, h). Quantification of the integrated CS density revealed that the cells contained more satellite (Fig. 3i). Furthermore, expanding these analyses for an evaluation of individual satellite characteristics confirmed that the satellites were significantly larger and covered an overall larger area (Fig. 3j). Thus, autophagy-deficient cells accumulate abnormal CS and exhibit a resulting dysregulation of centrosome composition.

### PCM1 and CEP131 interact with GABARAPs but not LC3B

**Fig. 4:**
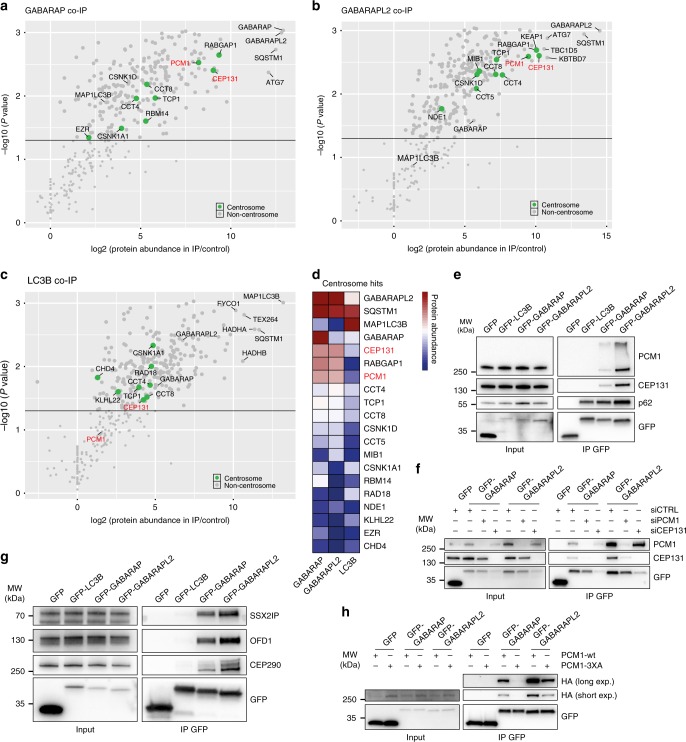
Centriolar satellite components interact with GABARAPs but not LC3B. **a**–**c** Scatter plots showing enrichment values (*x*-axis) and corresponding significance levels (*y*-axis) for proteins co-purifying with GFP-tagged GABARAP, GABARAPL2 or LC3B, *n* = 4. Centrosomal proteins are indicated in green, horizontal line indicates significance with threshold *P*-value < 0.05. **d** Heat map representing abundance of centrosomal proteins across co-IPs from A-C with *p* < 0.05 for at least one bait. Data are bait normalized to correct for differences in expression levels of bait proteins. **e** GFP-precipitation of lysates from inducible GFP-3xFLAG or GFP-tagged LC3B, GABARAP or GABARAPL2 MCF7 cells analyzed for co-precipitation of endogenous CEP131 and PCM1, *n* = 3. **f** GFP precipitation of GFP-3xFLAG, GFP-GABARAP or GFP-GABARAPL2 in U2OS cells treated with control, PCM1 or CEP131 siRNAs and blotted for co-precipitation of endogenous PCM1 and CEP131, *n* = 3. **g** GFP-precipitation of HEK293 lysates following co-transfection of GFP-3xFLAG or GFP-tagged GABARAP or GABARAPL2 with HA-PCM1-wt or HA-PCM1-3XA analyzed for co-precipitation of HA-tagged PCM1 variants, *n* = 3. **h** GFP-precipitation of GFP-3xFLAG or GFP-tagged LC3B, GABARAP or GABARAPL2 in HEK293 cells analyzed for co-precipitation of endogenous SSX2IP, OFD1 and CEP290, *n* = 3

To investigate if abnormal CSs could be attributed to autophagy regulation of specific CS-associated proteins, the interaction networks of selected ATG8 family members were analyzed. LC3B, GABARAP, and GABARAPL2 were chosen as representatives, as they are the most abundant ATG8s in our employed cell lines ([www.proteinatlas.org])^29^. For identification of ATG8 interaction partners, MCF7 cell lines (selected as they cope well with autophagy deficiency) stably expressing doxycycline-inducible GFP-tagged LC3B, GABARAP, GABARAPL2 or a control GFP-3xFLAG construct were used for GFP co-precipitation experiments, and the resulting precipitates were analyzed by liquid chromatography-mass spectrometry (LC-MS). The identified interaction partners were classified by their statistical significance and fold enrichment over the GFP control (Fig. 4a–c, and Supplementary Fig. 4A–C), and were evaluated for centrosome (Fig. 4a–c) or autophagy (Supplementary Fig. 4A–C) annotations. In addition, heat maps were generated for comparison of interactor abundance between the three ATG8 variants (Fig. 4d and Supplementary Fig. 4D–E). For a full list of identified proteins see Supplementary Data 1. As expected, a high number of autophagy-annotated proteins co-precipitated with all three ATG8 variants (Supplementary Fig. 4A–E, Supplementary Data 1), confirming the validity of our approach. Interestingly, among the most abundant and specific interaction partners of GABARAP and GABARAPL2, we found two CS components, CEP131^30^ and PCM1, neither of which were enriched in the LC3B precipitate (Fig. 4a–d, Supplementary Fig. 4E, Supplementary Data 1). In addition, the CS E3 ligase MIB1^31^ was identified as a weaker hit for GABARAPL2 (Fig. 4b, d). Notably, both PCM1 and CEP131 have previously been identified as ATG8-interactors^32,33^, supporting our MS results. Also identified as a strong centrosome-annotated hit was RABGAP1 (Fig. 4a–d), a GTPase-activating protein of RAB6^34^, that has been linked to autophagy regulation^35^. In our forthcoming analyses, we focused our attention on the well-established and most significantly enriched CS proteins PCM1 and CEP131.

Co-precipitation of PCM1 and CEP131 with GABARAP and GABARAPL2 but not LC3B was validated in ATG8-inducible MCF7 cells. Of note, both proteins displayed a preference for GABARAPL2 over GABARAP (Fig. 4e). In addition, PCM1 and CEP131 co-precipitated with endogenous GABARAP in HEK293 and MCF7 cells (Supplementary Fig. 4F, G).

To determine if CEP131 also showed dysregulation upon depletion of autophagy factors, the organization of CEP131 in CS was analyzed. Similarly, to PCM1 (see Fig. 3g–j), CEP131 formed large abnormal CS and string-like structures, resulting in an increased integrated density, larger CS and an overall larger satellite area (Supplementary Fig. 4H–K). This suggests a general disorganization of the CS and not only of individual CS components.

PCM1 and CEP131 are widely reported to interact^30,31^, indicating that they may be co-precipitated as a complex. To evaluate this possibility, a pull-down was performed upon depletion of PCM1 or CEP131. While PCM1 still interacted with GABARAP and GABARAPL2 after CEP131 depletion, CEP131 was lost from the precipitate upon silencing of PCM1 (Fig. 4f). Thus, CEP131 is indirectly co-precipitated with GABARAPs through PCM1. Accordingly, when increasing stringency in the pull-down procedure by varying salt and urea concentrations, CEP131 dissociated from GABARAP and GABARAPL2 before PCM1, suggesting a weaker association for CEP131 (Supplementary Fig. 4L, M).

Of note, the CS factor OFD1 has previously been reported to interact with ATG8 family members^32^. To determine if GABARAPs primarily interacted with the PCM1-CEP131 complex or the entire CS structure, additional well-established CS members SSX2IP^36^, OFD1^37^, and CEP290^38^ were evaluated for association with ATG8 proteins. Indeed, all three proteins interacted almost exclusively with GABARAP and GABARAPL2 and not with LC3B, with a preference for GABARAPL2 (Fig. 4g), as observed for PCM1 and CEP131.

The selectivity of PCM1 toward specific ATG8 proteins prompted us to investigate if the PCM1/GABARAP interaction was mediated through a LIR motif. To identify potential LIR motifs in PCM1 (PCM1 LIRs) we carried out sequence-based predictions. The identified motifs were ranked according to the position-specific scoring matrix (PSSM) scores provided by iLIR^39^ and consistent with disorder prediction by MobiDB^40^. Two motifs were identified with equally high scores, one of which, the motif 1961-EDFVKV-1966, was in agreement with the recently identified GABARAP-interacting motif (GIM) [W/F]-[V/I]-X2-V^41^, and a match for the PCM1 LIR recently experimentally validated^33^. In agreement with the results reported by Joachim et al.^33^, mutation of the PCM1 LIR from EDFVKV to a triple alanine mutant, EAAVKA (PCM1-3XA), abolished the interaction with GABARAP and reduced the binding to GABARAPL2 by ∼80% (Fig. 4h, Supplementary Fig. 4N), confirming that the interactions are LIR-dependent. To show direct physical interactions of the identified PCM1 LIR with the human ATG8s and to characterize affinity of these interactions, we performed isothermal titration calorimetry (ITC), by using a synthetic peptide spanning the PCM1 LIR sequence and representative human ATG8 analogs (GABARAP, GABARAPL2, and LC3B, see Supplementary Fig. 5; Supplementary Table 1 for details). In line with the co-precipitation experiments described above (see Fig. 4e), the ITC assays show that PCM1 LIR binds to LC3B with very low affinity (K_D_ 76 μM), while GABARAP-proteins exhibit more specific interactions with PCM1 LIR, with GABARAPL2 (K_D_ 3.2 μM) resulting as a stronger binder than GABARAP (K_D_ 6.4 μM).

**Fig. 5:**
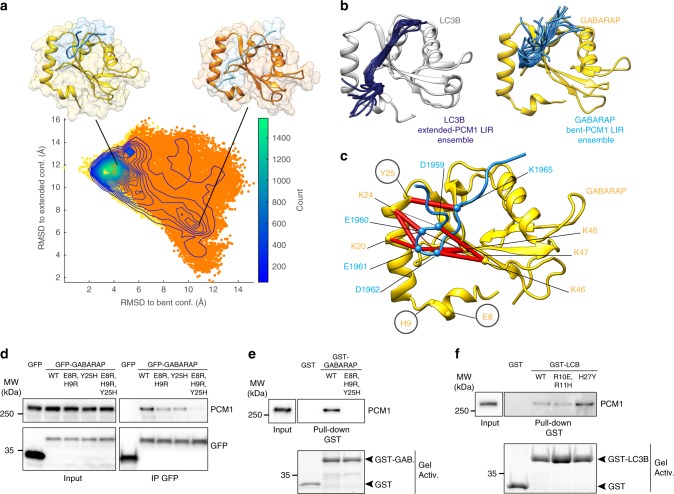
Structural analysis of GABARAP-PCM1 peptide complex. **a** Density plot for the conformations of PCM1 LIR in the GABARAP binding pocket, sampled during simulations starting from the bent (in yellow) and extended (in orange) conformations. The plot illustrates the similarity of the PCM1 LIR (light blue cartoon) conformations (measured as all-atom RMSD) with respect to each of the two initial structures for the simulations in complex with GABARAP (i.e., the bent and the extended conformation in yellow and orange cartoons, respectively). Starting from different conformations, the simulations of the PCM1 LIR into the GABARAP pocket converge on a similar ensemble of bent structures. **b** The common ensemble of bent structures of the PCM1 LIR peptide and ten representative conformations were isolated and are shown as a light blue cartoon. As a comparison, ten structures of the PCM1 LIR peptide from the simulations in complex with LC3B were reported and are indicated as dark blue cartoon. **c** Pairwise intermolecular contacts between residues of GABARAP and PCM1 LIR peptide were estimated in the selected ensemble. Contacts are represented as red cylinders connecting the Cα atoms of the pair of residues involved in the interaction. The radius of each cylinder is proportional to the occurrence of the contact, e.g. a contact presents in all structures of the selected ensemble has a radius of 0.5 Å. We here report the most important residues of GABARAP (yellow cartoon, E8, H9, K20, K46, K47, K48, and Y25) for the interaction between the 1959-DEED-1962 and K1965 of PCM1-LIR (light blue cartoon). The residues selected for experimental mutagenesis are circled and their Cα atoms are shown as spheres. **d** GFP-precipitation of GFP-3xFLAG or GFP-tagged GABARAP-wt or the indicated mutants in HEK293 cells analyzed for co-precipitation of endogenous PCM1, *n* = 2. **e** GST pull-down of GST, GST-GABARAP-wt or the indicated mutant analyzed for co-precipitation of endogenous PCM1, in HEK293 cells, *n* = 2. **f** GST pull-down of GST, GST-LC3B-wt or the indicated mutants analyzed for co-precipitation of endogenous PCM1, in HEK293 cells, *n* = 2

The determinants of binding specificity of LIR motifs to distinct ATG8s are not well-understood and could be different for each individual LIR. Thus, we used modeling and molecular dynamics (MD) simulations to identify the putative structural elements required for PCM1-GABARAP interaction. We focused on studying the PCM1-GABARAP interaction, since no experimental structures of GABARAPL2 in complex with a LIR motif are currently available. In our models, we used the 1955-SQKSDEED**FVKV**EDL-1969 peptide (LIR core region in **bold**), which comprises residues outside the LIR core region, thus accounting for potential distal effects. Interestingly, the results of our simulations show a bent and less extended conformation of the PCM1 LIR peptide in the GABARAP pocket, compared to that observed in the LC3B pocket (Fig. 5a, b). Indeed, the negatively-charged residue E8 of GABARAP has the potential to promote such a bent conformation through electrostatic repulsion with the PCM1 residues 1959-DEED-1962 (Fig. 5c). Also, once entrapped in the bent conformation, the PCM1 LIR may form a fluid electrostatic network with different lysine clusters (K20, K24, K46, K47 or K48) of GABARAP (Fig. 5c). Of note, the extended LIR conformation observed in the LC3B pocket could be favored by two positively charged residues (R10 and R11, corresponding - respectively - to E8 and H9 in GABARAP) interacting with the LIR N-terminal part. Also, an intermolecular hydrogen bond occurring between the K1965 of PCM1 and the Y25 of GABARAP may be crucial to the PCM1 selectivity for GABARAP, as already shown for another interaction^42^ (Fig. 5c), since it does not occur in our modeling relative to LC3B, in which Y25 corresponds to H27 (see [https://github.com/ELELAB/PCM1_LIR]).

Based on this model and in order to biochemically validate it, we generated two GABARAP mutants (E8R, H9R, and Y25H), in which selected residues were substituted with the corresponding LC3B residues; our intent was to push the LIR into a putative extended conformation, and destabilize the LIR-GABARAP interaction. As shown in Fig. 5d, both mutants partially disrupt the binding to PCM1, while the triple mutant (E8R, H9R, Y25H) almost abolishes it (Fig. 5d, e). By contrast, the H27Y mutation in LC3B increases the affinity of this ATG8 for PCM1, as shown in pull-down experiments (Fig. 5f). In line with these results, ITC experiments also revealed an effect of swapping LC3B/GABARAP mutations. Indeed, mutation of invariant Y25 in GABARAP to H reduces the affinity of PCM1 LIR recognition (Supplementary Fig. 6, left plots); instead, reciprocal mutation of H27 in LC3B to Y increases LC3B affinity to PCM1 LIR (Supplementary Fig. 6, right plots). Both mutants show the expected change in the binding enthalpy (see Supplementary Table 1), thus supporting the importance of the intermolecular hydrogen bond between PCM1 K1965 and the hydroxyl group of GABARAP Y25 for the observed binding-specificity (similar to the GABARAP-KBTBD6 LIR interaction)^43^. Of note, our data were further supported by a recent structural study published while our manuscript was under revision^44^.

In sum, we conclude that the observed PCM1 specificity toward GABARAP is achieved through intermolecular interactions involving (i) residues inside (K1965) and immediately upstream (1959-DEED-1962) the core PCM1 LIR, and (ii) specific polar (Y25) and negatively-charged (E8) residues in GABARAP.

### Centriolar satellites are autophagy substrates

**Fig. 6:**
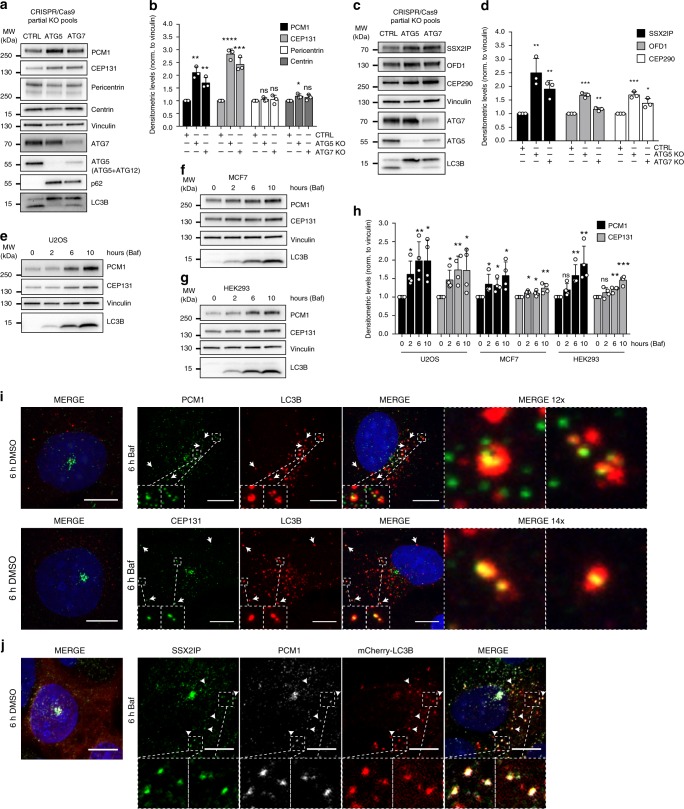
The CS are autophagy substrates. **a** MCF7 cell extracts of stable CRISPR/Cas9 non-targeting control (CTRL), ATG5- or ATG7-transfected partial knock-out pools immunoblotted for CS and centrosome proteins. Vinculin is used as loading control. **b** Densitometric quantification of CS and centrosome protein levels relative to vinculin, represented in (**a**). Columns represent the mean ± SD, *n* = 3, ns *P* > 0.05 *P ≤ 0.05, **P ≤ 0.01, ***P ≤ 0.001, ****P ≤ 0.0001. Unpaired Student’s *t*-test, two-tailed. **c** Stable CRISPR/Cas9 non-targeting control (CTRL), ATG5- or ATG7 partial knock-out MCF7 pools immunoblotted for CS proteins. Vinculin is used as loading control. **d** Densitometric quantification of CS protein levels relative to vinculin, represented in (**c**). Columns represent the mean ± SD, *n* = 3, *P ≤ 0.05, **P ≤ 0.01, ***P ≤ 0.001. Unpaired Student’s *t*-test, two-tailed. **e**–**g** Immunoblot of U2OS (**e**), MCF7 (**f**) or HEK293 (**g**) cells treated with Baf for the indicated times and immunoblotted for PCM1, CEP131, and vinculin as loading control. **h** Densitometric quantification of PCM1 and CEP131 immunoblots represented in (**e**–**g**). Columns represent the mean ± SD, *n* = 4, ns *P* > 0.05, *P ≤ 0.05, **P ≤ 0.01, ***P ≤ 0.001. Unpaired Student’s *t*-test, two-tailed. **i** Colocalization of LC3B with PCM1 or CEP131 in U2OS cells after 6 h of Baf treatment. Arrows indicate examples of colocalization. **j** Colocalization between SSX2IP, PCM1, and mCherry-LC3B in U2OS cells treated for 6 h with 200 nM Baf. Arrows indicate examples of colocalization. Scale bars, 10 µm. Source data are provided as a Source Data file

Prompted by the strong association between CS components and GABARAPs, combined with the striking accumulation of abnormal CS upon depletion of autophagy regulators, we investigated if CS proteins are autophagy substrates. To this end, stable MCF7 CRISPR/Cas9 ATG5 and ATG7 partial knock-out (KO) cell lines were employed. Both cell lines showed elevated protein levels of PCM1 and CEP131 compared to controls, while other dysregulated centrosome proteins, Pericentrin, and centrin, were not markedly affected (Fig. 6a, b). Similarly, SSX2IP, OFD1 and CEP290 also accumulated in autophagy-deficient cells (Fig. 6c, d), suggesting that CS are autophagy substrates. Further corroborating this, treatment with the lysosomal inhibitor bafilomycin A1 (Baf) caused a gradual accumulation of PCM1 and CEP131 in U2OS, MCF7 and HEK293 cells (Fig. 6e–h), revealing that autophagy governs a baseline turnover of CS factors. Reversely, autophagy induction by starvation gradually decreased the level of both PCM1 and CEP131, which was blocked by Baf treatment (Supplementary Fig. 7A–B), indicating that CS are also degraded by autophagy under autophagy-inducing conditions. Accordingly, the CS displayed a marked decrease in intensity, as visualized by PCM1 and CEP131 co-staining, after 6 hours of starvation (Supplementary Fig. 7C).

To study colocalization between CS components and autophagosomes, LC3B was employed as an autophagosome marker. Of note, LC3B localizes with GABARAP and GABARAPL2 on shared autophagosomes^45^. Upon Baf treatment clear co-localization was observed between PCM1 and CEP131 and the accumulated LC3B-positive autophagosomes, often at sites distant from the main CS cluster (Fig. 6i). Similar results were obtained for SSX2IP, OFD1, and CEP290 (Supplementary Fig. 7D), confirming autophagosomal engulfment of CS substrates and indicating that degradation takes place distantly from the centrosome. Importantly, SSX2IP and PCM1 co-localized with the same autophagosomes (Fig. 6j), further supporting that the CS are engulfed as a complex rather than as individual CS protein components. Also, co-localization between PCM1 and endogenous GABARAP or GABARAPL2-positive autophagosomes was observed (Supplementary Fig. 7E), as well as co-localization between PCM1 or CEP131 and autophagosomes marked by GFP-tagged versions of all three ATG8 variants (Supplementary Fig. 7F). Finally, ultrastructural analysis was performed by EM-immunogold detection of PCM1 in U2OS cells, showing numerous examples of sub-cellular co-localization between PCM1, CS and presumptive autophagosomal structures (Supplementary Fig. 7G–I).

Lysosomes are the final destination of the autophagy cargo. As expected for autophagy substrates, endogenous PCM1 and CEP131 localize in the lumen of swollen lysosomes (LAMP2-positive vesicles) of starved cells treated with the lysosomal inhibitor Baf (Supplementary Fig. 8A, B). Similarly, immunoaffinity purification of lysosomes showed an autophagy-induced enrichment of PCM1 and CEP131 at this organelle (Supplementary Fig. 8C, D).

The CS are highly dynamic structures and are reported to dissociate during mitosis^11^ and in response to a range of stress stimuli^31,46^. To investigate if autophagy plays a role in these responses, the CS status in autophagy-deficient cells was evaluated during mitosis and following treatment with anisomycin, an activating compound of the p38 kinase, reported to induce CS dissolution in response to stress^46^. The CS were dissolved in both control and autophagy-deficient mitotic cells, leaving comparable amounts of residual satellites (Supplementary Fig. 9A). This is in accordance with the reported inhibition of autophagy during mitosis^47^. Similarly, p38-induced CS dissolution occurred independently of autophagy status (Supplementary Fig. 9B–D). We, therefore, propose that autophagy governs a baseline CS turnover rather than a stimuli-induced rapid response. Of note, PCM1 could be observed co-localizing with LC3B in both G1, S and late G2 cells (Supplementary Fig. 9E), indicating that the degradation is not restricted to a specific phase of the cell cycle.

To further substantiate the link between CS accumulation and the occurrence of abnormal mitoses, the CS and mitotic response to CEP131 overexpression was investigated. CEP131 was employed as PCM1 overexpression frequently resulted in satellite dissolution (not shown). CEP131 formed aggregates when expressed above endogenous levels (Supplementary Fig. 10A, B), as previously reported^48^. These aggregates retained PCM1 and centrin (Supplementary Fig. 10A), indicating that CEP131 overexpression promoted the generation of abnormal satellite-like aggregates, mimicking a more severe form of the abnormal CS observed in autophagy-deficient cells. Importantly, CEP131 overexpression resulted in a high level of abnormal mitoses compared to overexpression of other dysregulated proteins, centrin or Pericentrin (Supplementary Fig. 10B, C). This implies that elevated levels of CS proteins are sufficient to dysregulate CS homeostasis and promote mitotic abnormalities.

### The PCM1 LIR motif is required for PCM1 autophagic clearance

**Fig. 7:**
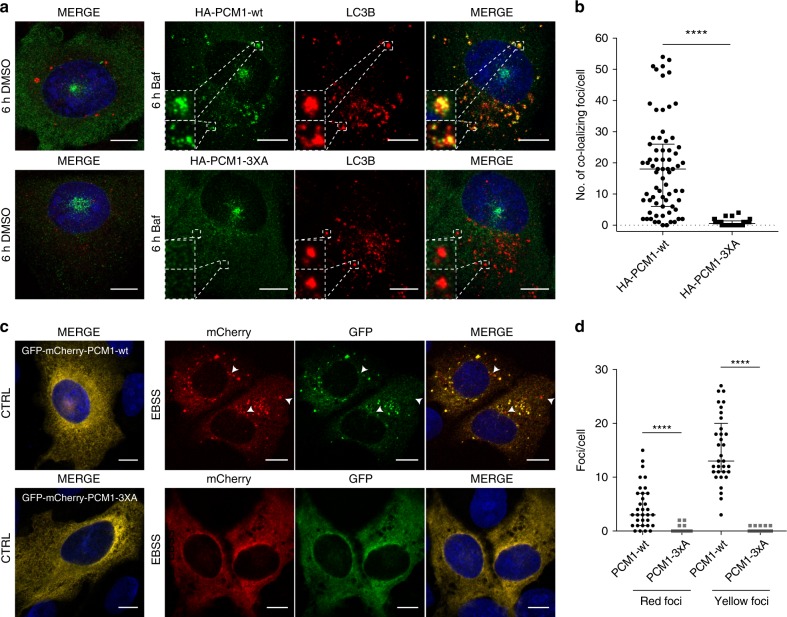
The PCM1 LIR domain is required for its autophagosomal engulfment. **a** Colocalization of LC3B with HA-PCM1-wt or HA-PCM1-3XA constructs in U2OS cells treated for 6 h with 200 nM Baf and stained for HA, LC3B and Hoechst33342. **b** Quantification of (**a**). The number of co-localizing foci per cell were quantified, *n* = 3 of each >20 cells. Bars represent medians and interquartile range. ****P ≤ 0.0001. Two-tailed Mann–Whitney test. **c** Traffic-light-assay of GFP-mCherry-PCM1 or GFP-mCherry-PCM1-3XA in U2OS cells following 2 h of EBSS treatment showing formation of yellow and red foci for PCM1, the latter indicating lysosomal localization. Arrows indicate examples of red foci. **d** Quantification of (**c**). The number of yellow and red foci per cell were quantified, *n* = 3 of each ≥10 cells. Bars represent medians and interquartile range. ****P ≤ 0.0001. Two-tailed Mann–Whitney test. Scale bars, 10 µm. Source data are provided as a Source Data file

The PCM1 LIR motif was previously shown to be required for PCM1 co-localization with autophagosomes^33^. To investigate the significance of the PCM1 LIR motif for the autophagic clearance of PCM1, PCM1-wt and PCM1-3XA were expressed in U2OS cells. Baf treatment resulted in a striking accumulation of PCM1-wt in LC3B-positive autophagosomes (Fig. 7a, b), similarly to endogenous PCM1 (Fig. 6i), while PCM1-3XA remained associated with the CS and diffusely in the cytosol (Fig. 7a, b). This strongly indicates that autophagosomal engulfment of PCM1 is mediated through a direct interaction with ATG8 proteins depending on the PCM1 LIR motif. Accordingly, the abnormal PCM1 CS in autophagy-deficient cells did not co-localize with p62 (Supplementary Fig. 10D). In addition, as PCM1 and CEP131 are reported to be ubiquitylated by the CS-resident E3 ubiquitin ligase MIB1^31^, we investigated the requirement of MIB1 for PCM1 degradation. Depletion of MIB1 did not affect Baf-induced accumulation or EBSS-induced degradation of PCM1 (Supplementary Fig. 10E–H), indicating that MIB1-mediated ubiquitylation is not required for autophagy-dependent PCM1 turnover.

As PCM1 has previously been linked to autophagy regulation^33^, a traffic-light-assay was employed to establish if autophagosome-associated PCM1 and CEP131 were transferred to lysosomes. GFP-mCherry-PCM1, but not PCM1-3XA, readily formed both yellow and red foci upon EBSS treatment (Fig. 7c, d), confirming that autophagosome-associated PCM1 is engulfed in autophagosomes and transferred to lysosomes. A co-staining for LC3B confirmed that the yellow foci co-localized with LC3B (Supplementary Fig. 10I), and thus represent autophagosome-associated PCM1.

Due to the aggregation-prone nature of CEP131, a co-staining against LC3B was performed to ensure that CEP131 aggregates associated with autophagosomes, like endogenous CEP131 (see Fig. 6i). This confirmed CEP131 co-localization with autophagosomes (Supplementary Fig. 10J). As PCM1, GFP-mCherry-CEP131 formed red foci upon EBSS treatment (Supplementary Fig. 10K), indicating that also CEP131 is transferred to lysosomes for degradation.

### PCM1 regulation and mitotic defects show GABARAP selectivity

**Fig. 8:**
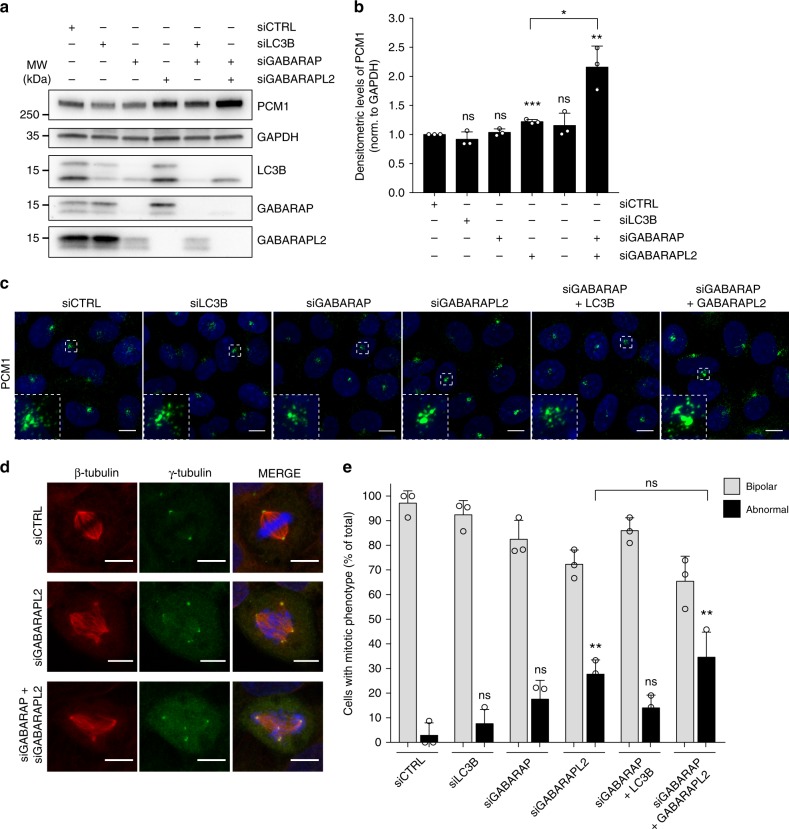
PCM1 accumulation and mitotic abnormalities show GABARAP selectivity. **a** Immunoblot of U2OS cell extracts following depletion of LC3B, GABARAP and GABARAPL2 individually or in combination as indicated showing levels of PCM1. GAPDH is used as loading control. **b** Densitometric quantification of PCM1 levels relative to GAPDH, represented in (**a**). Columns represent the mean ± SD, *n* = 3, ns > 0.05, **P ≤ 0.01, ***P ≤ 0.001. Unpaired Student’s *t*-test, two-tailed. **c** Representative images of PCM1-stained CS in U2OS cells depleted of the denoted ATG8 proteins. **d** Representative images of mitotic abnormalities in U2OS cells stained for γ-tubulin, β-tubulin and Hoechst33342 following depletion of the denoted ATG8 proteins. **e** Quantification of phenotype distribution in (**d**). Columns represent the mean ± SD, *n* = 3 of ≥20 cells, ns *P* > 0.05, **P ≤ 0.01. Unpaired Student’s *t*-test, two-tailed. Abnormal refers to cells exhibiting multipolar, diffuse bipolar or monopolar mitoses. Scale bars, 10 µm. Source data are provided as a Source Data file

Since PCM1 LIR is required for autophagosomal engulfment of PCM1 (see Fig. 7) and its interaction with GABARAPs (see Figs. 4h, 6 and ref. ^33^), we investigated if PCM1 accumulation and the formation of abnormal mitoses showed GABARAP selectivity. Interestingly, depletion of GABARAPL2 but not of LC3B or GABARAP resulted in accumulation of PCM1 (Fig. 8a, b), consistent with the stronger association observed for this ATG8 variant (see Fig. 4e). Furthermore, co-depletion of GABARAP and GABARAPL2 augmented the accumulation PCM1 while GABARAP and LC3B co-depletion did not affect PCM1, confirming the requirement of GABARAPs for regulation of PCM1 levels. Accordingly, only depletion of GABARAPL2 or combined depletion of GABARAP and GABARAPL2 resulted in the formation of abnormal CS (Fig. 8c) and significant increases in the formation of abnormal mitoses (Fig. 8d, e). Thus, PCM1 accumulation and mitotic abnormalities display GABARAP selectivity.

## Discussion

The list of cellular components identified as selective autophagy substrates continues to grow and adds to the complexity of autophagy-related pathologies. Here we show that CS proteins are targeted for autophagy by selective recognition of their main organizer PCM1, mediated through a GABARAP-specific C-terminal LIR motif. Interestingly, in recent years, an increasing number of substrate-resident autophagy receptors, that bridge the cellular compartment/component to which they localize and the autophagy machinery, have been identified^2^. Thus, extending this theme, PCM1 could be viewed as a specific CS receptor, and for this reason, we may consider the identified degradation pathway as a novel type of CS selective autophagy, i.e. doryphagy (from the Greek word *doryfóros* for satellite).

Whether the CS are recruited in the context of the centrosome or in the cytosol remains to be determined. We primarily observed co-localization between CS and autophagosomes distantly from the main CS aster (see Fig. 6i). However, as GABARAP was recently reported to co-localize with CS^33^, and several autophagy proteins have been observed in the vicinity of the centrosome^49^, we speculate that local autophagy regulators could mediate CS recruitment near the centrosome and promote their subsequent relocation for degradation. This, in principle, may function both at a baseline level or upon specific recognition and targeting of abnormal CS.

The selectivity of the PCM1-ATG8 interaction, PCM1 levels and mitotic abnormalities toward GABARAPs, and more specifically GABARAPL2, confers an additional level of regulation to the CS degradation pathway, and it is tempting to speculate that different ATG8s may provide specificity to the autophagy pathway in terms of substrate selectivity. Increasing our knowledge on the determinants of LIR motifs giving preference for specific ATG8 proteins may aid the distinction between their separate roles and the identification of functional LIRs in general. Here we suggest a putative contribution for the charged residues of the sequence DEED immediately upstream the PCM1 LIR in providing specificity for GABARAP together with the previously identified LIR (also termed GIM)^41^. Moreover, we identified some ATG8 determinants of binding specificity (see Fig. 5c–f). In addition, we are tempted to speculate that the emerging difference between LC3 and GABARAP pockets for binding the PCM1 LIR may also reside in the GABARAP capability to induce a LIR bent conformation, thanks to both electrostatic and polar interactions (see Fig. 5a–c). While such a bent conformation is occasionally observed in the unit cells from the crystallographic structure of the PCM1 LIR bound to GABARAP (PDB entry 6HYM^44^), its existence needs to be experimentally proven.

The accumulation of highly abnormal CS upon autophagy factor depletion (see Fig. 3g–j, Supplementary Fig. 4H–K) implies that autophagy plays a central role in maintaining appropriate satellite levels and organization. How autophagy deficiency affects CS functionality is, however, difficult to discern, as the CS regulate centrosome composition in a highly complex manner, promoting the centrosomal recruitment of some components while sequestering and retaining others^9^. We hypothesize that the large abnormal CS in autophagy-deficient cells are over aggregated, and consequently, impaired in their fusion/dissociation dynamics. Indeed, accumulation of centrosome proteins (e.g. centrin) in CS has previously been interpreted as an indication of impaired trafficking through the satellites^50^. Thus, the observed CS accumulation of centrin, CEP63 and Pericentrin (see Fig. 3f, Supplementary Figs. 2E, 3E), that all require CS for their centrosomal targeting^10,51^, suggests impaired CS dynamics. Nonetheless, the increase in centrosomal Pericentrin (see Fig. 3a–c) may indicate exaggerated recruitment, which would imply that the accumulated CS are not entirely dysfunctional. The mitotic centrosome fragmentation resulting from this CS dysregulation, highlights the significance of proper CS function for maintaining centrosome integrity. Corroborating the link between CS dysfunction and aberrant mitosis, are several reports showing that manipulation of CS proteins, including CEP131, results in mitotic centrosome defects and, in particular, centrosome fragmentation^19,30,36,52,53^.

While our findings prompted us to focus our attention on the role of autophagic CS regulation for cell division, proper CS function must be expected to influence all aspects of centrosome functionality, e.g. primary cilium (PC) formation and centrosome cycle progression. Accordingly, autophagy was previously reported to regulate PC assembly by degrading the ciliary protein IFT20 and the CS component OFD1^32,49^, corroborating a role for doryphagy in ciliogenesis. Furthermore, we speculate that stress-induced autophagy may potentially modify the CS for stress regulation of centrosome and cell cycle progression. Indeed, we observe a marked decrease in CS levels during starvation (see Supplementary Fig. 7A, B). Recent reports have uncovered that the CS are highly stress-responsive structures and that they dissociate following a wide range of insults^31,46^. We did not observe any effect of autophagy status on these responses (see Supplementary Fig. 9B–D). Doryphagy may thus represent an additional CS-modifying pathway during stress. The functional significance of stress-induced CS-remodeling remains to be determined, but it could represent a way to halt the centrosome cycle in a coordinate manner with the cell cycle, as several steps depend on CS-delivered centrosome factors^9^. Indeed, cross-talk between autophagy and cell cycle arrest pathways are well-documented^54^. In this context, it is noteworthy that depletion of PCM1 is reported to induce a p38-p53-p21-dependent cell cycle arrest^55^. We may, therefore, speculate that autophagy-mediated PCM1 and CS degradation could facilitate cell cycle arrest. The functional interplay between the CS and autophagy during stress induction is an interesting aspect for further study.

Intriguingly, the PC also relays external cues for autophagy induction through autophagy regulators present at the PC basal body (centrosome)^49^, implying that PC/autophagy regulation is bi-directional. CS/autophagy regulation also appears to function in a bi-directional manner, as it was recently published that PCM1 promotes GABARAP stabilization and consequently regulates autophagy^33^. PCM1-mediated GABARAP stabilization requires the PCM1-LIR motif;^33^ thus, it would be interesting to understand how the switch between GABARAP mediated PCM1-degradation and PCM1-mediated GABARAP stabilization occurs.

Autophagy deficiency has in several instances been linked to chromosomal instability^3,13,56^. While the multifunctional nature of autophagy makes it difficult to pinpoint a single cause, it is interesting to speculate that centrosome dysfunction could be a key contributing factor. Indeed, centrosome abnormalities have been noted in a number of autophagy-deficient cell systems^12,13^. In accordance with our findings showing a high level of postmitotic cell death in autophagy-defective cells (see Fig. 2a, b), centrosome abnormalities and polyploidy in Beclin 1-deficient immortalized baby mouse kidney epithelial cells were shown to strongly increase in cells with defective apoptosis due to Bcl-2 expression^13^. This suggests that, also in this system, centrosome dysfunction is partially suppressed by the demise of highly abnormal cells, implying that a degree of compensation is to be expected. In addition, centrosome defects often activate p53-dependent responses^5,55^, implying that accumulation of centrosome abnormalities relies on the ability to overcome such stress regulation. This may explain why many stable autophagy-deficient systems do not display evident growth retardation. Nonetheless, stable autophagy-deficient systems were shown to exhibit centrosome accumulation^12^. While we do not observe these abnormalities, excess centrin-containing CS and increased centrosomal Pericentrin have previously been proposed as facilitators of centrosome accumulation^57,58^, indicating a potential link between our and previously reported centrosomal defects.

From the centrosome viewpoint, it is well-established that centrosome abnormalities, including both structural and numerical changes, are an intrinsic feature of many types of cancer^5,6^. The origin of these defects, however, is not well-understood. The CS may be particularly interesting in this context, due to their key impact on centrosome composition, reported here and by others^9^. Intriguingly, increased levels of CEP131 were recently reported in both hepatocellular carcinoma and breast carcinoma^59,60^. We thus speculate that CS functionality, and their regulation by doryphagy, could play an underestimated role in tumorigenesis.

## Methods

### Cell lines and cell culture

All cell lines were grown at 37 °C in a humidified incubator containing 5% CO_2_.

U2OS osteosarcoma cells were purchased from the American Type Culture Collection (ATCC) and cultured in Dulbecco’s modified eagle’s medium (DMEM) GlutaMAX^TM^ (Gibco) supplemented with 10% fetal bovine serum (FBS) (Gibco) and antibiotics. U2OS mRFP-α-tubulin H2B-GFP cells (mixed population) were provided by L. Lanzetti^61^, and were cultured as described above supplemented with neomycin (500 µg/ml). U2OS GFP-centrin cells were generated by transfection of GFP-centrin in U2OS cells and subsequent G418 selection. The cells were subsequently cultured in DMEM supplemented with 500 µg/ml G418. HEK293 cells were cultured in DMEM GlutaMAX^TM^ (Gibco) supplemented with 10% FBS (Gibco) and antibiotics. MCF7 wild type and GFP-tagged doxycycline-inducible cell lines were grown in Roswell Park Memorial Institute (RPMI) GlutaMAX^TM^ (Gibco) medium supplemented with 10 % FBS (Gibco) and antibiotics. For induction of GFP-tagged ATG8 proteins 500 ng/ml doxycycline was added 24 hours prior to collection. For generation of CRISPR/Cas9 KO cell lines MCF7-eGFP-LC3 cells^62^ were used. The cells were grown in RPMI 1640 medium with 6 % FBS and antibiotics and were created by standard lentiviral transduction procedure using lentiCRISPR v2 constructs (non-targeting control, ATG5 or ATG7) obtained from the lab of Kevin Ryan (Beatson Institute for Cancer Research, Glasgow, UK)^63^.

### Transfections and treatments

Transient transfections were performed using GeneJuice transfection reagent (Merck-Millipore) for U2OS cells or polyethylenimine linear MW 25,000 (PEI) for HEK293 cells according to the manufacturers’ protocols. siRNA transfections were carried out using Lipofectamine RNAiMAX (Invitrogen) as described by the manufacturer.

BafilomycinA1 (Santa Cruz Biotechnology) was used in a concentration of 200 nM.

For induction of starvation, Earle’s Balanced Salt Solution (EBSS) (Sigma-Aldrich) was used.

The ULK1 inhibitor SBI-0206965 (Sigma-Aldrich) was used in a concentration of 5 µM.

### Constructs and siRNAs

All constructs are described in Supplementary Table 2, and the primers used for cloning/mutagenesis are listed in Supplementary Table 3.

Sequences for RNA interference are as follows:

Control (CTRL): ON-TARGETplus non-targeting control siRNA # 3 Dharmacon

siULK1: 5’-CCCUUUGCGUUAUAUUGUA-3’

siATG7: 5’-CAGUGGAUCUAAAUCUCAAACUGAU-3’

siPCM1: 5'-GGUUUUAACUAAUUAUGGA-3'

siCEP131: 5’-GCUAACAACAGGAGCAACA-3’

siATG5: Santa Cruz Biotechnology, sc-41445, siRNA pool.

siGABARAP: 5’-GGUCAGUUCUACUUCUUGA-3’

siGABARAPL2: 5’-UGGGCUAGGUGCACCGUAA-3’

siLC3B: 5’-CUCCCUAAGAGGAUCUUUAUU-3’

siMIB1: 5’-GGAUAAAGAUGGUGAUAGA-3’

### Time-lapse Imaging

For time-lapse imaging, cells were grown in glass-bottomed dishes (WillCo-dish; Willcowells) coated with 0.5% gelatin in PBS. RNAi was achieved by reverse and forward transfections on 2 consecutive days. Time-lapse imaging was performed on a Leica AF6000LX fluorescent workstation. The cells were placed onto a sample stage within an incubator chamber set at 37 °C in an atmosphere of 5% CO_2_ and 20% humidity 1 h before acquisition start. Z-stacks were captured with a ×40 objective for both channels every 5 min for 10 h beginning 48 h after the initial silencing. Lamp intensity was kept at a minimum to avoid phototoxicity. Automated acquisition of 5 different fields for each sample was performed using a high-precision motorized stage. Deconvolution and Z-stack projections were generated with the LAS AF Leica Application Suite software (Leica). For longer imaging (>10 h), acquisition was limited to the GFP channel in combination with DIC to further limit phototoxicity.

### Immunofluorescence (IF) and microscopy

In preparation for immunofluorescence analyses, cells were grown on plastic coverslips coated with 0.5% gelatin in PBS. Cells were fixed in ice-cold MeOH for 3 min at −20 °C and washed 3 times in PBS. For cell cycle analyses and microtubule regrowth assays cells were fixed in 4% formaldehyde for 10 min at room temperature (RT). For LAMP2 colocalization cells were fixed in 4 % formaldehyde followed by ice-cold MeOH. Permeabilization was performed in PBS plus 0.2% triton X-100 for 2 min at RT, followed by 30 min blocking in blocking buffer (PBS plus 5% FBS, 1% BSA and 0.3% triton X-100). The slides were incubated with primary antibodies in blocking buffer for 1 h at RT and washed 3 times in PBS plus 0.25% BSA and 0.1% triton X-100. Then the slides were incubated with the appropriate combination of secondary antibodies conjugated to Alexa Fluor 488, Alexa Fluor 568 or Alexa Fluor 647 (Life Technologies) diluted in PBS plus 5% FBS, 0.25% BSA and 0.1% triton X-100 for 1 h at RT. DNA was stained using Hoechst33342 (ThermoFisher Scientific, H3570, 1:1000 in PBS) or DAPI (Sigma Aldrich, D9542, 1:1000 in PBS) and finally, the slides were mounted in fluorescence mounting medium (Dako). Immunofluorescence analyses were carried out at laser scanning confocal microscopes (LSM700 and LSM800, Carl Zeiss A/S). For LAMP2 colocalization, 2 consecutive Z-stacks (pinhole = 0.7 μm; distance between planes = 0.2 μm) have been acquired and projected using the Max. Intensity projection. Given the size of the lysosomes (which are enlarged in our samples because of the Baf treatment), this approach allowed us to better visualize the single lysosomes as circular structures. Intensity profile plots have been calculated using the RGB profiler plugin of the ImageJ software.

### IF quantification

For quantification of mitotic, prophase and interphase phenotypes, fields or individual cells were randomly selected for analysis in the microscope and images acquired. After acquisition, the images were analyzed and the cells were scored in the denoted phenotype categories.

For quantification of fluorescence intensity or centrosome area, random fields were selected for acquisition on a confocal microscope and Z-stacks of 0.2 µm were acquired using standardized settings. Quantification of average pixel intensity was performed on average intensity projections using ImageJ software. For centrosome area quantification a common threshold was set to distinguish centrosomes from background and the size of individual centrosomes measured using ImageJ software.

Quantification of integrated density and centriolar satellite characteristics was performed on random fields acquired as 0.2 µm Z-stacks by confocal microscopy using standardized settings. In ImageJ software z-stacks were subjected to sum projection and a threshold was set to identify centriolar satellites from the background. Integrated density was calculated as (mean fluorescence intensity*total area) − (background fluorescence intensity*total area). The integrated density, mean fluorescence intensity, average object size, object number and total area were measured on a single cell basis.

### Immunoblotting

Cell lysates were prepared with triton lysis buffer (10 mM Tris/HCl pH 7.4, 150 mM NaCl, 1 % Triton X-100) for 30 min. on ice or with whole-cell lysis buffer (50 mM Tris-HCl pH 6.8, 10% Glycerol, 2% SDS) for 5 min at 95 °C for better dissolution of aggregates. Protein extracts were quantified using the DC protein assay (Bio-Rad), and denatured in NuPAGE® LDS Sample Buffer (Life technologies). Proteins were separated on acrylamide gradient gels (Bio-Rad) and blotted onto Polyvinylidene difluoride (PVDF) membranes (Bio-Rad) using the Trans-Blot turbo system (Bio-Rad). Blocking was performed in 5% non-fat dry milk in PBS plus 0.1 % Tween-20. Membranes were incubated in primary antibodies in 2% non-fat dry milk in PBS plus 0.1% Tween-20 at 4 °C overnight followed by incubation in secondary horseradish-peroxidase (HRP)-conjugated antibodies (Bio-Rad) (1:5000) for 1 hour at RT. Secondary antibody detection was achieved using Amersham ECL Prime (GE Healthcare). For quantification, densitometric levels were measured using Image Lab software. Uncropped files of all western blots are reported in the Supplementary Information, as Supplementary Fig. 11.

### Antibodies

Primary antibodies were as follows; ATG5 Cell Signaling Technology, 12994S, WB 1:1000), ATG7 (Cell Signaling Technology, 8558S, WB 1:1000), β-actin (Novus Biologicals, NB600-501, WB 1:5000), β-tubulin (Sigma-Aldrich, T4026, IF 1:200), centrin (a gift from I. Cheeseman^64^, Whitehead Institute for Biomedical, Research, Cambridge, USA, IF 1:1000), CEP63 (Proteintech, 16268-1-AP, IF 1:200), CEP131 (Bethyl, A301-425A, WB 1:1000; Abcam, ab84864, IF 1:300; Abcam, ab99379, WB 1:1000, IF 1:500), CEP290 (Abcam, ab84870, IF 1:200, WB 1:1000), CPAP (Proteintech, 11517-1-AP, IF 1:200), DIC 1/2 (Santa Cruz Biotechnology, sc-13524, WB 1:2000), EG5 (BD Transduction Laboratories, 611186, WB 1:2000), GABARAP (Abgent, AP1821a, WB 1:1000; Cell Signaling Technology, 13733S, WB 1:1000, IF 1:100), GABARAPL2 (Abcam, ab126607, WB 1:1000, IF 1:100), GAPDH (Merck-Millipore, CB1001, 1:20,000), GFP (Santa Cruz Biotechnology, sc-8334, WB 1:1000), γ-tubulin (Sigma-Aldrich, T3559, IF 1:200; Sigma-Aldrich, T5326, IF 1:250), HA (Sigma-Aldrich, H3663, WB 1:1000, IF 1:200), Histone H3 (Abcam, ab201456, WB 1:2000), LAMP2 (Abcam, ab25631, WB 1:2000; Developmental Studies Hybridoma Bank University of Iowa, H4B4, IF 1:100), LC3B (Cell Signaling Technology, 3868S, WB 1:1000, IF 1:200), LC3B (Cell Signaling Technology, 2775S, WB 1:1000), MIB1 (Novus Biologicals, NBP1-95846, WB 1:1000), NEDD1 (Santa Cruz Biotechnology, sc-100961, IF 1:200), NuMA (Abcam, ab36999, IF 1:200), OFD1 (Sigma-Aldrich, HPA031103, IF 1:200, WB 1:1000), p62/SQSTM1 (MBL International, PM045, WB 1:2000), p150Glued (BD Biosciences, 610473, WB 1:2000), PCM1 (Bethyl Laboratories, A301-150A, WB 1:1000, IF 1:500, EM 1:20; Santa Cruz Biotechnology, sc-398365, IF 1:200; Santa Cruz Biotechnology, sc-50164, IF 1:200), Pericentrin (Abcam, ab4448, WB 1:1000, IF 1:200), SSX2IP (Sigma-Aldrich, HPA027306, IF 1:200, WB 1:1000), ULK1 (Cell Signaling Technology, 8054, WB 1:1000), VDAC (Cell Signaling Technology, 12454 S, WB 1:1000), vinculin (Sigma-Aldrich, V4505, WB 1:2000).

### Cell cycle analyses

U2OS cells were plated on plastic coverslips and treated with control, ULK1, ATG7 or ATG5 siRNAs. 48 h after the silencing was initiated, the cells were incubated with 10 µM EdU (5-ethynyl-20-deoxyuridine) for 30 min and fixed in 4% formaldehyde. Subsequently, detection of EdU was performed with the Click-it EdU kit (Invitrogen), and the slides were counterstained with Hoechst33342. For each condition, 30 non-overlapping images of 3 independent experiments were acquired using the Scan^R screening station (Olympus). At least 1000 cells were processed using Scan^R Analysis software (Olympus) and Spotfire software (TIBCO). Bar graphs were prepared in Graph Pad Prism, *n* = 3.

### Microtubule regrowth assay

To depolymerize microtubules, U2OS cells were incubated with ice-cold media on ice for 1 h at 4 °C. To induce regrowth, the cold media was exchanged for 37 °C media and cells fixed in 4% formaldehyde at the indicated time-points. The cells were subsequently stained for β-tubulin for evaluation of microtubule nucleation and organization.

### Traffic-light-assay

EGFP-mCherry-PCM1 or EGFP-mCherry-PCM1-3XA were expressed in U2OS cells. Before fixation, the cells were incubated for 2 h in EBSS in order to induce autophagy. Random cells were acquired in Z-stacks by confocal microscopy for subsequent quantification of the number of yellow and red foci.

### Identification of cells in specific cell cycle phases

S-phase cells were identified by EdU staining. Cells were treated with Bafilomycin for 2 h and EdU for 30 min and fixed in ice-cold 100% MeOH. EdU staining was performed using the Click-iT® EdU Alexa Fluor® Imaging Kit (Thermo Fisher Scientific), according to the manufacturer’s protocol.

To identify G1 or G2 phase cells, stable U2OS GFP-centrin cells were treated with Bafilomycin 2 h prior to fixation in ice-cold 100% MeOH. G1 cells were identified as having one centrosome with two centrioles and late G2 phase cells as having two separated centrosomes each with two centrioles.

For all conditions, cells were counter-stained for PCM1 and LC3B and cells belonging to S-phase, G1-phase or G2 phase were identified by confocal microscopy. Bafilomycin treatment was required due to the low level of autophagosomes in the standard U2OS culture and was kept at a minimum length to limit the risk of cells changing cycle during treatment.

### Pull-down and co-immunoprecipitation (coIP) experiments

Dox-inducible MCF7 cell lines carrying GFP-3xFLAG, GFP-LC3B, GFP-GABARAP or GFP-GABARAPL2 were induced with 500 nM doxycycline 24 h prior to collection. Cells were lysed in standard IP lysis buffer (50 mM HEPES pH 7.4, 100 mM NaCl, 0.5% Triton-X100) containing protease and phosphatase inhibitors. Lysates were incubated with GFP-trap MA beads for 1 h at 4 °C for precipitation of GFP-tagged constructs. The beads were then washed 5 times in lysis buffer and the precipitates were eluted in 1.5× Laemmli sample buffer for 8 min at 95 °C and resolved by SDS-PAGE and western blotting.

For immunoprecipitations using transient transfection of inducible GFP-tagged constructs, induction was performed using 200 ng/ml doxycycline for 24 h and the pull-down performed as described above.

For pull-downs performed with increasing NaCl and urea concentrations, the beads were separated in equal aliquots following 1 h incubation with the lysates. All aliquots were washed 1× in standard IP lysis buffer and then 2 × 5 min in the following buffers: Standard IP lysis buffer, standard IP lysis buffer containing 500 mM NaCl, 500 mM NaCl + 1 M urea or 500 mM NaCl + 2 M urea. Subsequently, all aliquots were washed 1× in standard IP lysis buffer and eluted and analyzed as described above.

For immunoprecipitation of endogenous GABARAP, cells were lysed in standard IP lysis buffer supplemented with protease and phosphatase inhibitors. The clarified lysates were used for immunoprecipitation with the indicated antibodies overnight at 4 °C. Dynabeads Protein G were then added to collect the immunocomplexes. The beads were washed 3 times with standard IP lysis buffer and eluted with 2× Laemmli sample buffer at 95 °C for 10 min and analyzed as above.

### CoIP liquid chromatography-mass spectrometry (LC-MS)

MCF-7 cells stably expressing GFP-tagged GABARAP, GABARAPL2, LC3B or a control construct (GFP-3xFLAG) were grown in full medium, and 24 h prior to collection, protein expression was induced with doxycycline. Cells were collected and pelleted in ice-cold PBS. Cell pellets were lysed in standard IP lysis buffer, sonicated using Branson tip sonicator (settings: 2 × 15 s, amplitude 10%) and clarified by centrifugation (16000 × *g*, 10 min at 4 °C). Co-IPs were performed in biological quadruplicates using one 15 cm plate per experiment. Clarified lysates were incubated for 90 min with GFP-trap MA beads (Chromotek) and the beads were subjected to three washes in standard IP lysis buffer and followed by two washes with lysis buffer w/o detergent. Proteins were eluted with trypsin in 50 mM TAEB buffer. Reduced and alkylated peptides were immobilized and purified on C18 stage-tips (Pierce) and subjected to LC-MS analysis.

Tryptic peptides were identified by LC-MS using an EASY-nLC 1000 (Thermo Scientific) coupled to a Q Exactive HF (Thermo Scientific) equipped with a nanoelectrospray ion source. Peptides were separated on an in-house packed column of ReproSil-Pur C18-AQ, 3 µm resin (Dr Maisch, GmbH) using a 60-min gradient of solvent A (0.5% acetic acid) and solvent B (80% acetonitrile in 0.5% acetic acid) and a flow of 250 nL/min. The mass spectrometer was operated in positive ion mode with a top 12 data-dependent acquisition, a resolution of 60,000 (at 400 *m/z*), a scan range of 300–1700 *m/z* and an AGC target of 3e6 for the MS survey. MS/MS was performed at a scan range of 200–2000 *m/z* using a resolution of 60,000 (at 400 *m/z*), an AGC target of 1e5, an intensity threshold of 4.5e4 and an isolation window of 1.2 *m/z*. Further parameters included an exclusion time of 45 s and a maximum injection time for survey and MS/MS of 15 ms and 110 ms, respectively.

Protein identification and quantification were conducted by processing raw files obtained from LC-MS analysis using the MaxQuant software^65^ version 1.5.2.8 at default settings. Peak lists were searched against the human UniProt database with internal MaxQuant engine (Andromeda). The raw MaxQuant output data can be found in Table S1A. Statistical analysis was conducted for each biological quadruplicate in R using samr package and protein hits detected with at less than two unique peptides from further analysis. Median values of peptide intensities were calculated for each replicate and pseudo intensity counts of 1 × 10^6^ were added for median values equal to zero to calculate fold enrichment over background. Enrichment was calculated relative to control GFP-3xFLAG co-IP and indicated as ‘log2 fold change’. Significantly enriched proteins are indicated in Supplementary Data 1B, 1C and 1D. Scatter plots were made using ggplot R package and centrosome and autophagy gene ontology terms were extracted for AmiGo2 (geneontology.org)^66^.

### GST pull-down

The constructs encoding for GST-fused LC3B, GABARAP, and their respective mutants were expressed in E. coli BL21 (DE3) cells, and the expression of the fusion proteins was induced by addition of 0.5 mM IPTG at 37  °C over-night. Cells were lysed by sonication in lysis buffer (20 mM Tris-HCl pH 7.5, 10 mM EDTA, 5 mM EGTA, 150 mM NaCl) and Glutathione Sepharose 4B beads (GE Healthcare) were used to precipitate the GST-fused proteins. Fusion protein-bound beads (5 μl and 15 μl for the GABARAP and LC3B pull-downs, respectively) were used directly in GST pull-down assays. HEK293 (400 μg and 800 μg of total proteins for the GABARAP and LC3B pull-downs, respectively) were lysed in lysis buffer (50 mM TRIS, pH 7.5, 150 mM NaCI, 1 mM EDTA, 0.5% Triton X-100, supplemented with protease and phosphatase inhibitors). Lysates were cleared by centrifugation at 16,000 × *g* for 10 min, and incubated with GST fusion protein-loaded beads for 2 h, at 4 °C. Beads were then washed four times in lysis buffer, resuspended in NuPAGE® LDS Sample Buffer (Life Technologies) supplemented with 1% β-Mercaptoethanol and boiled. Supernatants were loaded on 4–15% TGX Stain-Free protein gels (Biorad) for SDS-PAGE and the immunoblot was performed as described above. GST and GST-fused proteins were visualized by gel activation (Stain-Free Imaging Technology, Biorad), as a substitute of the Coomassie Blue staining.

### Immunoaffinity purification of lysosomes

Cell homogenates were incubated with rabbit anti-LAMP1 antibody (Abcam, ab24170). The immunoprecipitates were collected by using magnetic MicroBeads coupled to goat anti-rabbit antibody (Miltenyi Biotec, 130-048-602) and MS Columns (Miltenyi Biotec, 130–042–201) equipped on OctoMACS^TM^ Separator (Miltenyi Biotec, 130-042-109).

### PCM1 LIR prediction

Prediction of LIR motifs was carried out using the iLIR database^39^, we then verified if the motifs were located in predicted disordered regions by a consensus method implemented in MobiDB^40^. We retained the two motifs with highest scores for PCM1 (555-STWNEV-560, 1961-EDFVKV-1966) of which 1961-EDFVKV-1966 was chosen for further evaluation.

### Molecular modeling and MD simulations

Generation of starting structure for MD simulations: We used Modeller version 9.15^67^ to generate structural models of the PCM1 LIR motif in complex with LC3B and GABARAP. For the modeling of LC3B in complex with PCM1, we used the Xray structure of the p62 LIR motif in complex with LC3B (PDB entry 2ZJD)^68^. To model the complex between GABARAP and PCM1, we used two different templates: (i) the X-ray structure of K1 peptide (PDB entry 3D32)^69^ and (ii) of KBTBD6 peptide (PDB entry 4XC2)^43^ in complex with GABARAP. We selected these two templates since they allow us to investigate different conformations of the LIR in the GABARAP pocket, using a bent (3D32) and extended (4XC2) conformation, respectively.

We generated 1000 binding poses with Modeller. We then carried out structural clustering with the linkage algorithm, using as a metric for structural similarity the Root Mean Square Deviation (RMSD) of all the atoms of the LIR peptides upon superimposition of the ensemble with THESEUS^70^. We selected the average structure from the most populated cluster (i.e., the conformer with the lowest RMSD to the other ones in the same cluster) and we used each of them as a starting structure for all-atom MD simulations.

MD simulations: We carried out 250-ns all-atom MD simulations in explicit solvent for each of the selected models. The MD simulations were performed with Gromacs version 5.1^71^ with the protein force field CHARMM22*^72^ and the TIPS3P water model^73^.

Each system was solvated in a dodecahedral box with a minimum distance between protein and box edge of 16 Å and charged ions were added to set a concentration of 150 mM NaCl and equilibrated before productive MD runs. We calculated the intermolecular contacts with the CONtact Analysis (CONAN) tool using default parameters used in the original publication^74^. We estimated, in each simulation of the GABARAP-PCM1 complexes, the RMSD of the PCM1 LIR peptide with respect to each of the two initial structures for the simulation, fitting on the GABARAP protein atoms. We then used the two RMSD profiles as reaction coordinate to describe the conformations sampled during the simulations. We estimate the associated density distribution, to isolate structures samples by both the simulations (in the range of 3.5–5 Å and 10.5–13.5 Å for the RMSD with respect to the bent and the extended conformation, respectively), for a total of 13278 structures. We extracted a comparable ensemble of 13742 structures from the simulations of LC3B in complex with PCM1 by filtering the trajectory every two frames.

### ITC and NMR samples preparation

For ITC experiments, LC3 and GABARAP proteins were obtained based on the protocols described elsewhere^75^. Human PCM1 LIR-contained peptide (1958-SDEEDFVKVEDLPLKLTIY-1976, core LIR is underlined) was purchased from GenScript Inc (N.J., USA). Before experiments, all proteins and peptide were equilibrated with a buffer containing 50 mM Tris-HCl, pH 7.4, 100 mM NaCl; and supplied with 5 mM protease inhibitors cocktail.

### Isothermal titration microcalorimetry (ITC)

The ITC experiments were performed at 25 °C using a MicroCal VP-ITC microcalorimeter (Malvern Instruments Ltd., UK). The PCM LIR peptide at concentrations of 0.4 mM was titrated into 0.025 mM LC3 and GABARAP proteins in 21 steps. The ITC data were analyzed with the ITC-Origin 7.0 software in assumption of a “one-site” binding model. The proteins and peptides concentrations were calculated from the UV-absorption at 280 nm by Nanodrop spectrophotometer (Thermo Fisher Scientific, DE, USA).

### CS analyses of mitotic cells and upon anisomycin-treatment

U2OS cells were treated with control, ULK1 or ATG7 siRNAs. For stress-induced CS dissolution, the p38-activating compound anisomycin was used in a concentration of 1 µg/ml for 2 h. For IF staining of centriolar satellites in combination with γ-tubulin, cells were fixed in ice-cold 1:1 methanol/acetone, dried for 15 min at room temperature (RT) and rehydrated in PBS. Coverslips were incubated with primary antibodies diluted in DMEM for 1 h at RT. Subsequently, coverslips were stained with secondary antibodies (Alexa Fluor 488 and 568, Life Technologies) for 30 min. Finally, coverslips were mounted with Vectashield mounting medium (Vector Laboratories) containing nuclear stain 4,6-diamidino-2-phenylindole (DAPI). For fixation of IF samples of centriolar satellites without γ-tubulin, cells were fixed in 4% formaldehyde (VWR), permeabilized with PBS containing 0.2% Triton X-100 for 5 min and immunostained as above. Images were acquired with an LSM780 confocal microscope (Carl Zeiss Microimaging Inc.) mounted on a Zeiss-Axiovert 100 M equipped with a Plan-Apochromatic ×40/1.3 oil immersion objective. Image acquisition and analysis were carried out with ZEN2010 (Zeiss). Presence of centriolar satellites was quantified by assessing individual cells for presence of a cluster of PCM1 or CEP131 in direct vicinity of the centrosome in both axial (x, y) and lateral (z) directions.

### Electron microscopy

Cells (U2OS) for immuno-EM were fixed with 4% formaldehyde and 0.1% glutaraldehyde in 0.1 M PHEM (60 mM Pipes, 25 mM Hepes, 10 mM EGTA and 2 mM MgCl_2_, pH 7.4) over-night, scraped from the culture dish in 1% gelatin/PBS, pelleted (3000 rpm, 5 min) and resuspended in 12% gelatin/PBS. After pelleting at 10,000 rpm for 5 min the gelatin was solidified on ice and small sample cubes prepared for infiltration with 2.3 M sucrose over-night at 4 °C. The next day, the samples were mounted on sample holders, frozen in lN_2_ and sectioned (70–90 nm sections) at −110 °C on a Leica Ultracut equipped with cryo chamber. Sections were picked up with a 50:50 solution of 2.3 M sucrose/2% methyl cellulose, transferred to formvar/carbon-coated grids and labeled with anti-PCM1 antibody followed by 10 nm proteinA-gold (UMC, Utrecht, Netherlands). Samples were observed in a JEOL-JEM 1230 at 80 kV and images recorded with a Morada CCD camera (Olympus, Germany).

### Statistics and reproducibility

Statistical analyses were performed with Prism software (GraphPad Software). Error bars in bar plots represent means ± SD. Each experiment was performed ≥3 independent times with similar results. All representative experimental findings were verified in ≥3 independent experiments. In Fig. 5d–f, instead, *n* = 2. Exact number of repeats and the number of analyzed cells per repeat is indicated in figure legends. For quantification of differences in mitotic duration, the two-tailed Mann–Whitney test was used to compare medians. Unless otherwise stated, the remaining analyses were performed using the two-tailed Student’s *t*-test to compare differences between means. Statistical significance was defined as: ns *P* > 0.05, *P ≤ 0.05, **P ≤ 0.01, ***P ≤ 0.001, ****P ≤ 0.0001.

### Reporting summary

Further information on research design is available in the Nature Research Reporting Summary linked to this article.

## Supplementary information

Supplementary Information

Supplementary Data 1

Movie 1A

Movie 1B

Movie 2A

Movie 2B

Movie 3A

Movie 3B

Movie 4A

Movie 4B

Movie 5A

Movie 5B

Movie 6A

Movie 6B

Reporting Summary

Source Data

## Data Availability

The source data underlying all Main and Supplementary Figures are provided as a Source Data file. A Reporting Summary for this article is available as a Supplementary Information file. The raw ITC data are available from the corresponding author upon reasonable request. The mass spectrometry proteomics data have been deposited to the ProteomeXchange Consortium via the PRIDE partner repository with the dataset identifier PXD014829 [https://www.ebi.ac.uk/pride/archive/projects/PXD014829].

## Code availability

All the computer scripts and source codes used to generate and analyse the results presented in the manuscript are publicly available at E. Papaleo Group Github repository [https://github.com/ELELAB/PCM1_LIR].
