# Supplementary material for: Selective autophagy maintains centrosome integrity and accurate mitosis by turnover of centriolar satellites
Source: Nat Commun. 2019 Sep 13;10:4176. doi: 10.1038/s41467-019-12094-9 (PMC6744468; doi:10.1038/s41467-019-12094-9)
Supplement: Supplementary file 1 — Supplementary Information [file 41467_2019_12094_MOESM1_ESM.pdf]

Selective autophagy maintains centrosome integrity and accurate mitosis by turnover of centriolar satellites

Holdgaard *et al.*

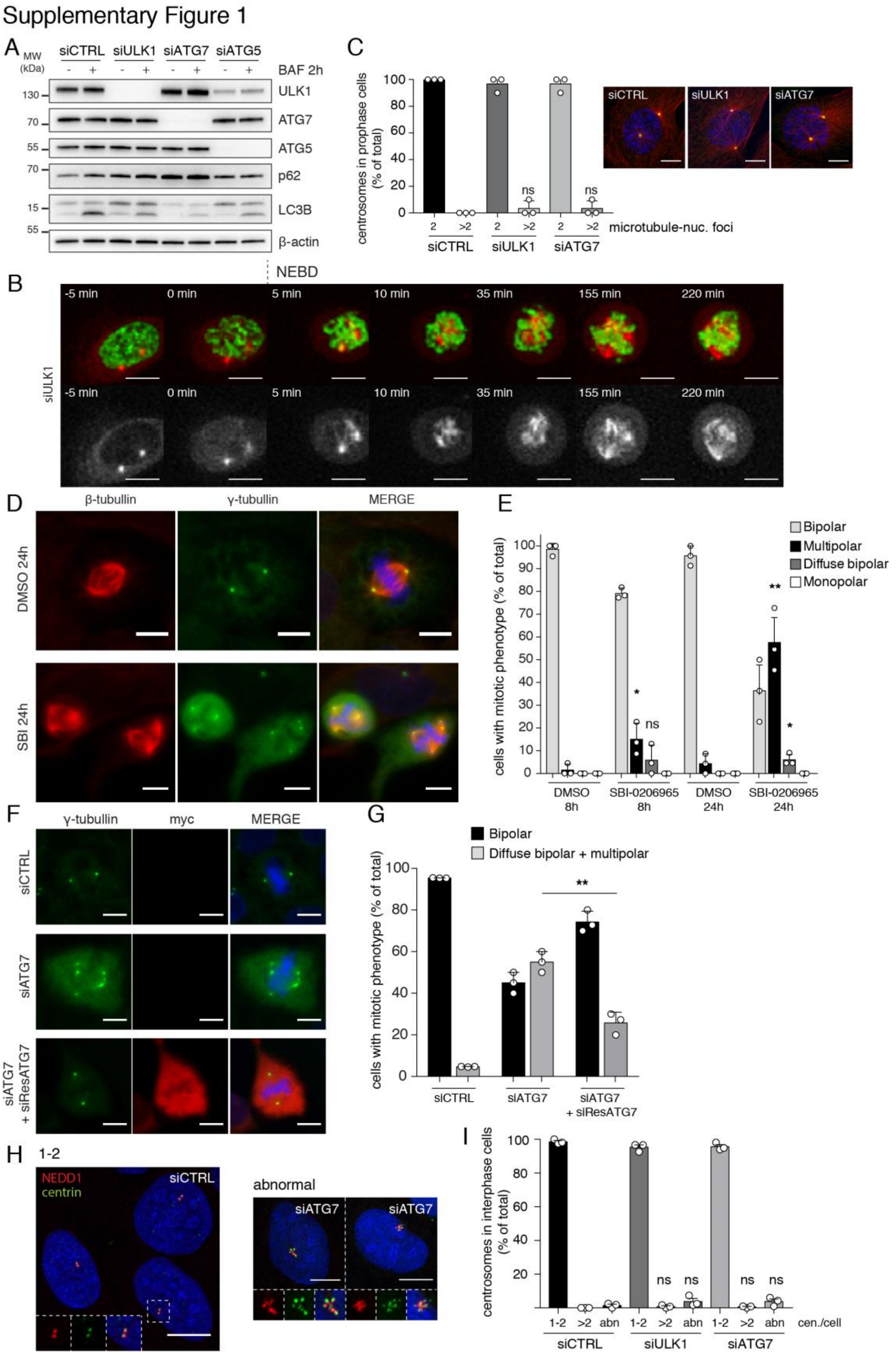

Supplementary Figure 1. Analysis of centrosome and mitotic phenotypes upon autophagy inhibition. (A) Immunoblot showing efficiency of knock-down of ULK1, ATG7 or ATG5 in U2OS cells treated with Baf for evaluation of autophagy flux by immunoblotting for LC3B and p62. **(B)** Time-lapse imaging of stable U2OS mRFP-α-tubulin H2B-GFP cells depleted of ULK1. Images were acquired every 5 min for 10 hours. Time after NEBD is indicated. **(C)** Centrosome status in prophase cells treated with control, ULK1 or ATG7 siRNAs, stained for β-tubulin, γ-tubulin and Hoechst33342. Prophase cells were scored as having 2 well-defined centrosomes (normal) or > 2 γ-tubulin foci (abnormal). Columns represent means ± SD, n = 3 of ≥ 10 cells. ns P > 0.05. Unpaired Student's *t*-test, two-tailed. **(D)** Abnormal mitoses in U2OS cells treated with 5 µM of the ULK1 inhibitor SBI-0206965 for the indicated time points, stained for γ-tubulin, β-tubulin and Hoechst33342. **(E)** Quantification of phenotype distribution in D. Columns represent the mean ± SD, n = 3 of ≥ 20 cells, ns P > 0.05, * P ≤ 0.05, ** P ≤ 0.01. Unpaired Student's *t*-test, two-tailed. **(F)** Representative images of ATG7 rescue experiments. U2OS were depleted of ATG7 +/- re-introduction of siRNA-resistant ATG7. **(G)** Quantification of mitotic phenotypes in F. Columns represent the mean ± SD, n = 3 of ≥ 10 cells, ** P ≤ 0.01. Unpaired Student's *t*-test, two-tailed. **(H)** Images for quantification of centrosome number/cell as evaluated by centrin and NEDD1 co-staining following depletion of ULK1 or ATG7. **(I)** Quantification of centrosome number per cell. Cells were scored as normal (1-2 centrosomes), centrosome accumulation (>2 centrosomes) or abnormal (aggregate-like centrin and NEDD1 structures). Columns represent means ± SD, n = 3 of ≥ 100 cells. ns P > 0.05. Unpaired Student's *t*-test, two-tailed. Scale bars, 10 µm. Source data are provided as a Source Data file.

Supplementary Figure 2

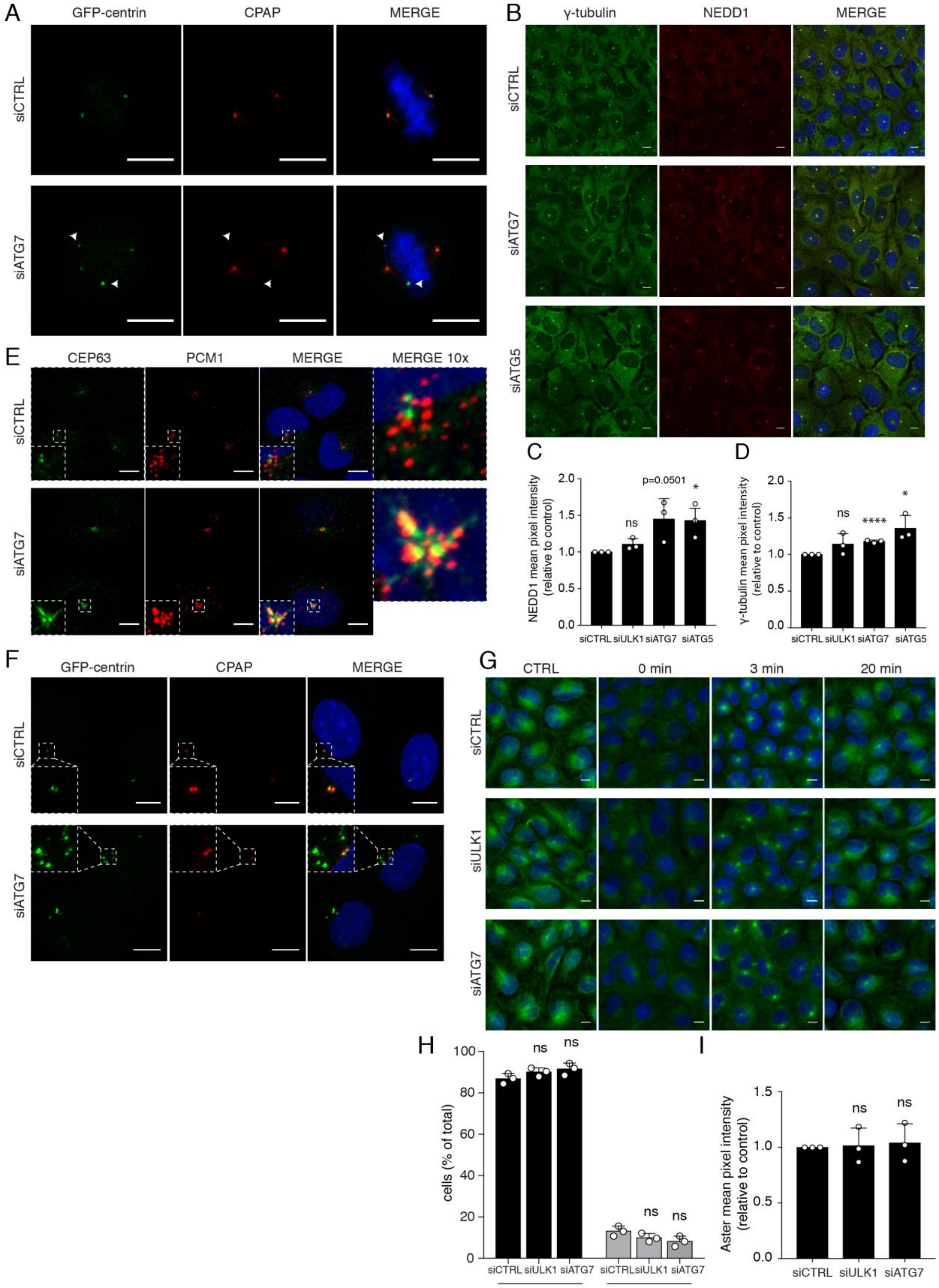

Supplementary Figure 2. Analysis of centrosomal proteins in autophagy deficient cells. (A) U2OS stable GFP-centrin expressing cells were treated with control or ATG7 siRNA and stained for the centriole marker CPAP and Hoechst33342. Arrows indicate acentrosomal centrin foci. **(B)** NEDD1 and γ-tubulin co-staining of U2OS cells transfected with control, ATG7 or ATG5 siRNAs. **(C-D)** Quantification of B. Columns represent mean fluorescence intensity of NEDD1 (C) or γ-tubulin (D) ± SD, n = 3 of > 60 cells. ns P > 0.05, * P ≤ 0.05, **** P ≤ 0.0001. Unpaired Student's *t*-test, two-tailed. **(E)** Co-localization between CEP63 and PCM1 in U2OS cells transfected with control or ATG7 siRNAs. **(F)** Stable GFP-centrin expressing U2OS cells treated with control or ATG7 siRNA and stained for the centriole marker CPAP and Hoechst33342. **(G)** Representative images of microtubule regrowth assays in U2OS treated with control, ULK1 or ATG7 siRNAs. Microtubules were depolymerized by cold treatment and regrowth induced by addition of 37°C DMEM. Cells were fixed at the indicated time points and stained for β-tubulin and Hoechst33342. **(H-I)** Quantification of G. Columns represent the mean frequency of aster formation (H) and mean aster fluorescence intensity (I) at 3 min after regrowth ± SD, n = 3 of ≥ 100 cells. ns P > 0.05, * P ≤ 0.05, **** P ≤ 0.0001. Unpaired Student's *t*-test, two-tailed. Scale bars, 10 µm. Source data are provided as a Source Data file.

Supplementary Figure 3

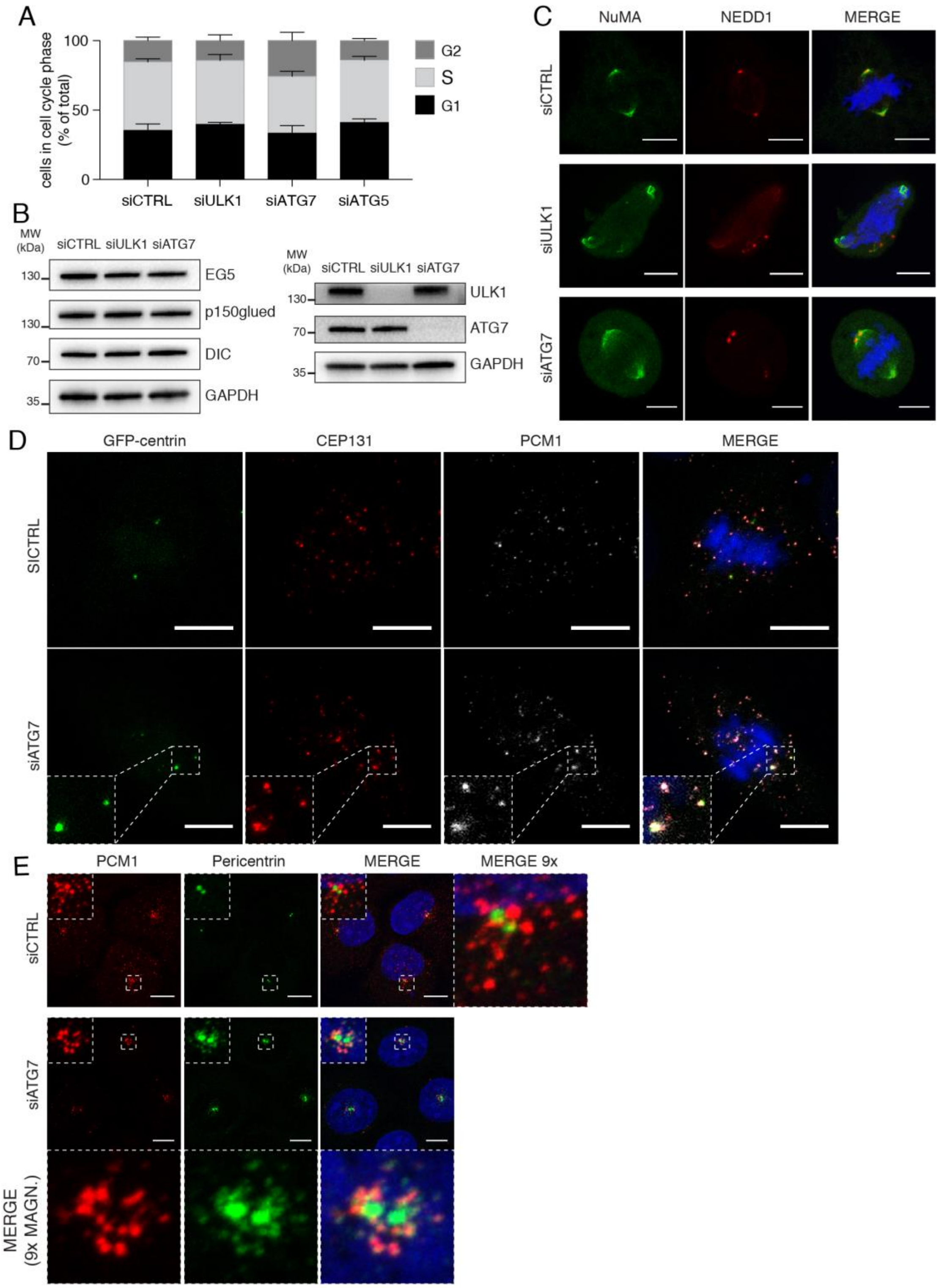

Supplementary Figure 3. Cell cycle, motor proteins and dynein-mediated transport are not affected by autophagy. (A) Cell cycle distribution in U2OS cells depleted of ULK1, ATG7 or ATG5. The cells were incubated with EdU for 30 min and counterstained with Hoechst33342 for evaluation of cell cycle distribution. Columns represent means ± S.D. n = 3 independent experiments. **(B)** Immunoblot showing protein levels of key motor proteins upon transfection with control, ULK1 or ATG7 siRNAs. **(C)** Immunofluorescence images showing localization of NuMA in U2OS cells treated with control, ULK1 or ATG7 siRNAs. **(D)** Stable GFP-centrin expressing U2OS cells treated with control or ATG7 siRNA and stained for CEP131, PCM1 and Hoechst33342 for evaluation of co-localization in mitotic cells. Scale bars, 10 µm. **(E)** Co-localization between Pericentrin and PCM1 in U2OS cells transfected with control or ATG7 siRNA. Scale bars, 10 µm. Source data are provided as a Source Data file.

Supplementary Figure 4

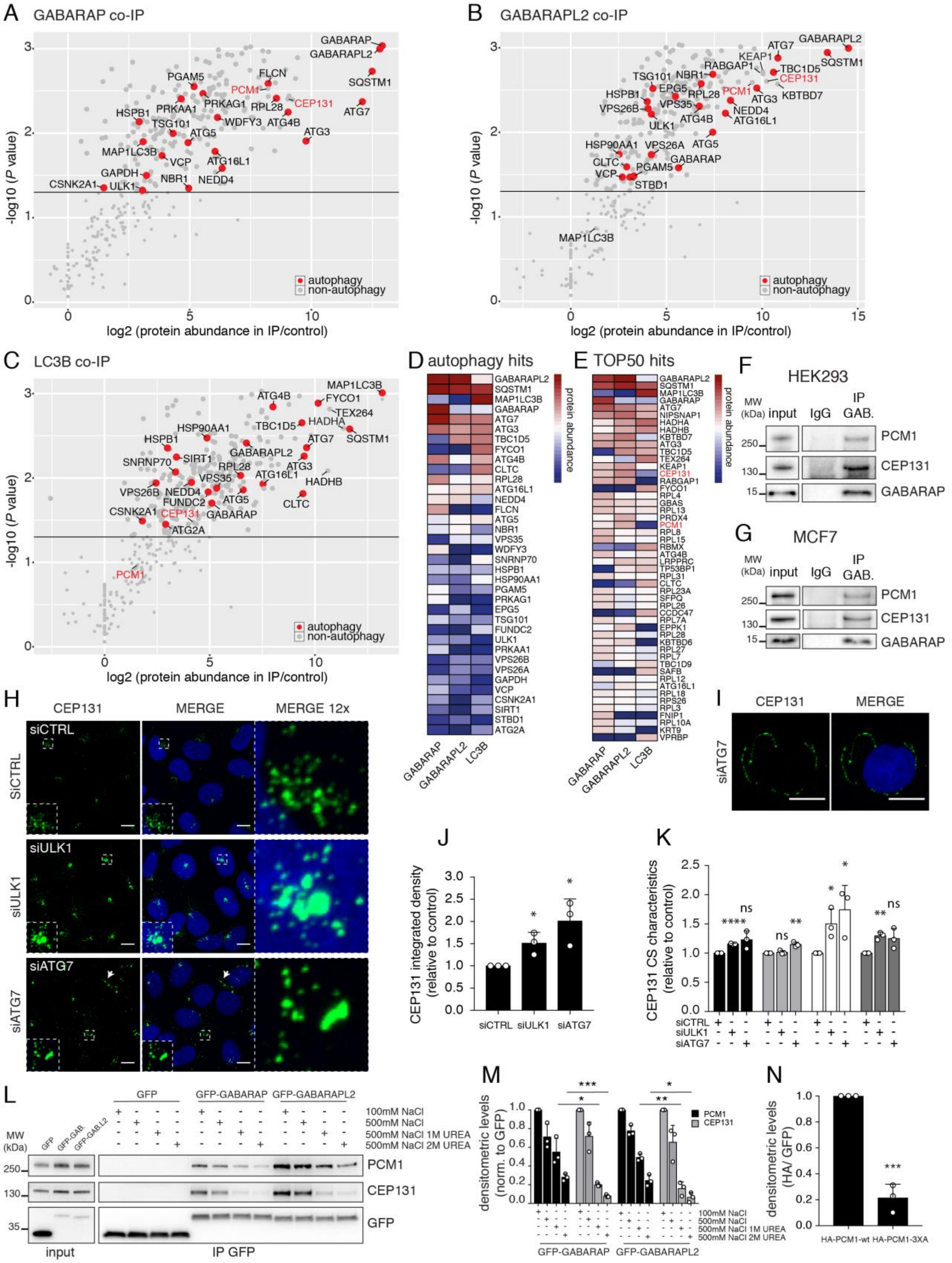

Supplementary Figure 4. ATG8 interactome and characterization of PCM1 and CEP131 as GABARAPs interactors. (A-C) Scatter plots showing enrichment values (x-axis) and corresponding significance levels (y-axis) for proteins co-purifying with GFP-tagged GABARAP, GABARAPL2 and LC3B (n = 4), as in Fig. 4A-C. Autophagy-associated proteins are indicated in red, horizontal line indicates significance with threshold P value < 0.05. **(D-E)** Heat maps representing abundance of autophagy-associated proteins (D) and top 50 identified proteins (E) identified in co-IPs from (A-C). Data are bait normalized to correct for differences in expression levels of bait proteins. **(F-G)** Immunoprecipitation of endogenous GABARAP in HEK293 (F) and MCF7 (G) cells blotted for co-precipitation of endogenous PCM1 and CEP131. **(H)** CEP131 distribution in U2OS cells after treatment with control, ULK1 or ATG7 siRNAs. Arrows indicate string-like CEP131. Scale bars, 10 µm. **(I)** String-like distribution of CEP131 in ATG7-silenced U2OS cells. **(J)** Quantification of integrated density of experiments represented in H. Columns represent the mean ± SD, n = 3 of > 50 cells. * P ≤ 0.05. Unpaired Student's *t*-test, two-tailed. **(K)** Quantification of CS characteristics of experiments represented in H. Columns represent the mean ± SD, n = 3 of > 50 cells. ns P > 0.05, * P ≤ 0.05, ** P ≤ 0.01, **** P ≤ 0.0001. Unpaired Student's *t*-test, two-tailed. **(L)** GFP-precipitation of HEK293 lysates following transfection and dox-induction of GFP-3xFLAG or GFP-tagged GABARAP or GABARAPL2 using washes with increasing concentrations of NaCl and urea, n = 3. **(M)** Densitometric quantification of L, normalized to the level of precipitated bait protein. Columns represent mean ± SD, n = 3, * P ≤ 0.05, ** P ≤ 0.01, *** P ≤ 0.001. Unpaired Student's *t*-test, two-tailed. **(N)** Densitometric quantification of HA-PCM1-wt and HA-PCM1-3XA co-precipitated with GFP-GABARAPL2, normalized to the densitometric level of the precipitated bait. Columns represent mean ± SD, n = 3, *** P ≤ 0.001. Unpaired Student's *t*-test, two-tailed. Source data are provided as a Source Data file.

Supplementary Figure 5

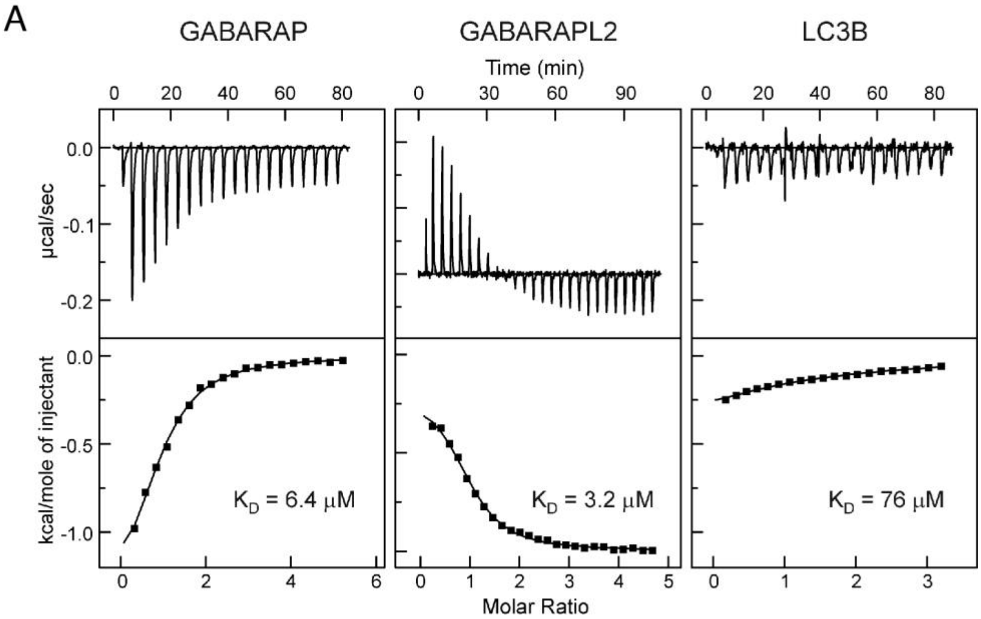

Supplementary Figure 5. PCM1 LIR interaction with human ATG8 proteins show GABARAP-subfamily preference. Interaction between PCM1 LIR peptide (residues 1958-1976) and selected human ATG8 proteins (GABARAP, GABARAPL2 and LC3B) investigated by isothermal titrational calorimetry (ITC). The ITC experiments showed that the PCM1 LIR binds to LC3B proteins with lowest affinity. K_D value for this interaction (76 μM) is up to 100 times higher in comparison to that for canonical p62 LIR and is equivalent to the affinities of non-phosphorylated OPTN LIR and Nix peptides1[1, 2]. GABARAP proteins showed more specific interactions to PCM1 LIR, with GABARAPL2 resulting as a strongest binder with K_D of 3.2 μM (6.4 μM for GABARAP). All tested human ATG8 analogues revealed a small favorable (negative) enthalpy contribution (ΔH of ~1-2 kcal/mol); for GABARAPL2, the binding enthalpy is positive (+1 kcal/mol), making entropical factors (like buried hydrophobic surfaces) the most pronounced driving force of interaction. The top diagrams in each ITC plot display the raw measurements, while the bottom diagrams show the integrated heat per titration step. Best fit is presented as a solid line. Measured K_D values are indicated for each interaction.

Supplementary Figure 6

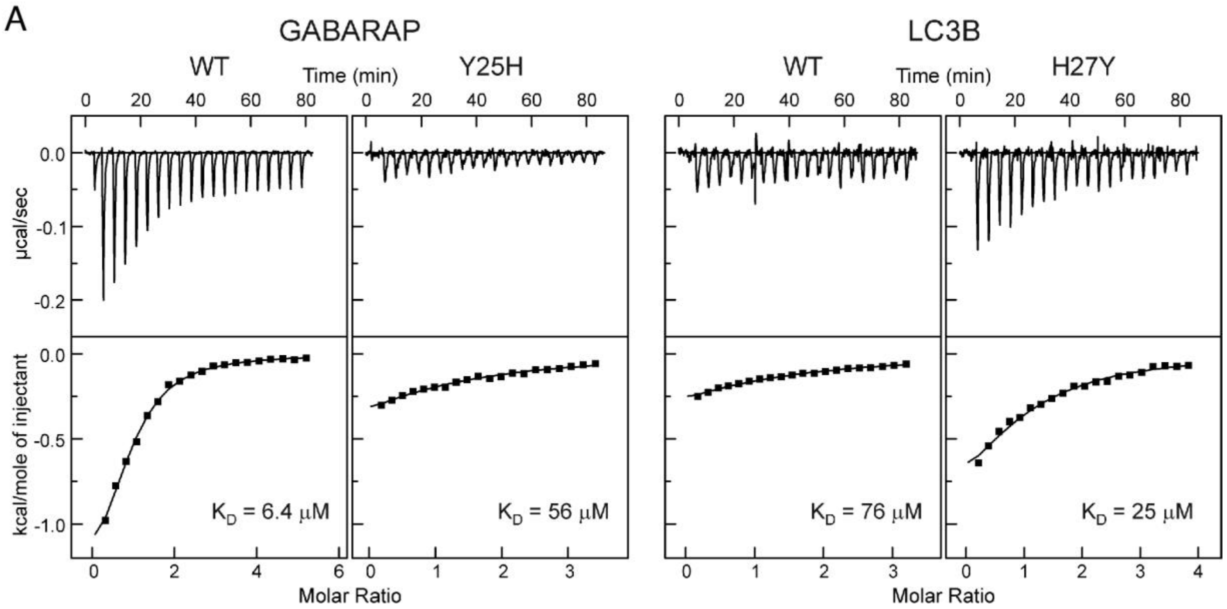

Supplementary Figure 6. Y25 of GABARAP is a key determinant of the sub-family specificity to PCM1 LIR. GABARAP Y25 and LC3B H27 are important for the PCM1 LIR specificity, as shown by experiment of ITC titrations of the PCM1 LIR peptide into GABARAP-wt and Y25H mutant (left plots) and into LC3B-wt and H27Y mutant (right plots). The top diagrams in each ITC plot display the raw measurements and the bottom diagrams show the integrated heat per titration step. Best fit is presented as a solid line. Measured K_D values are indicated for each interaction.

Supplementary Figure 7a

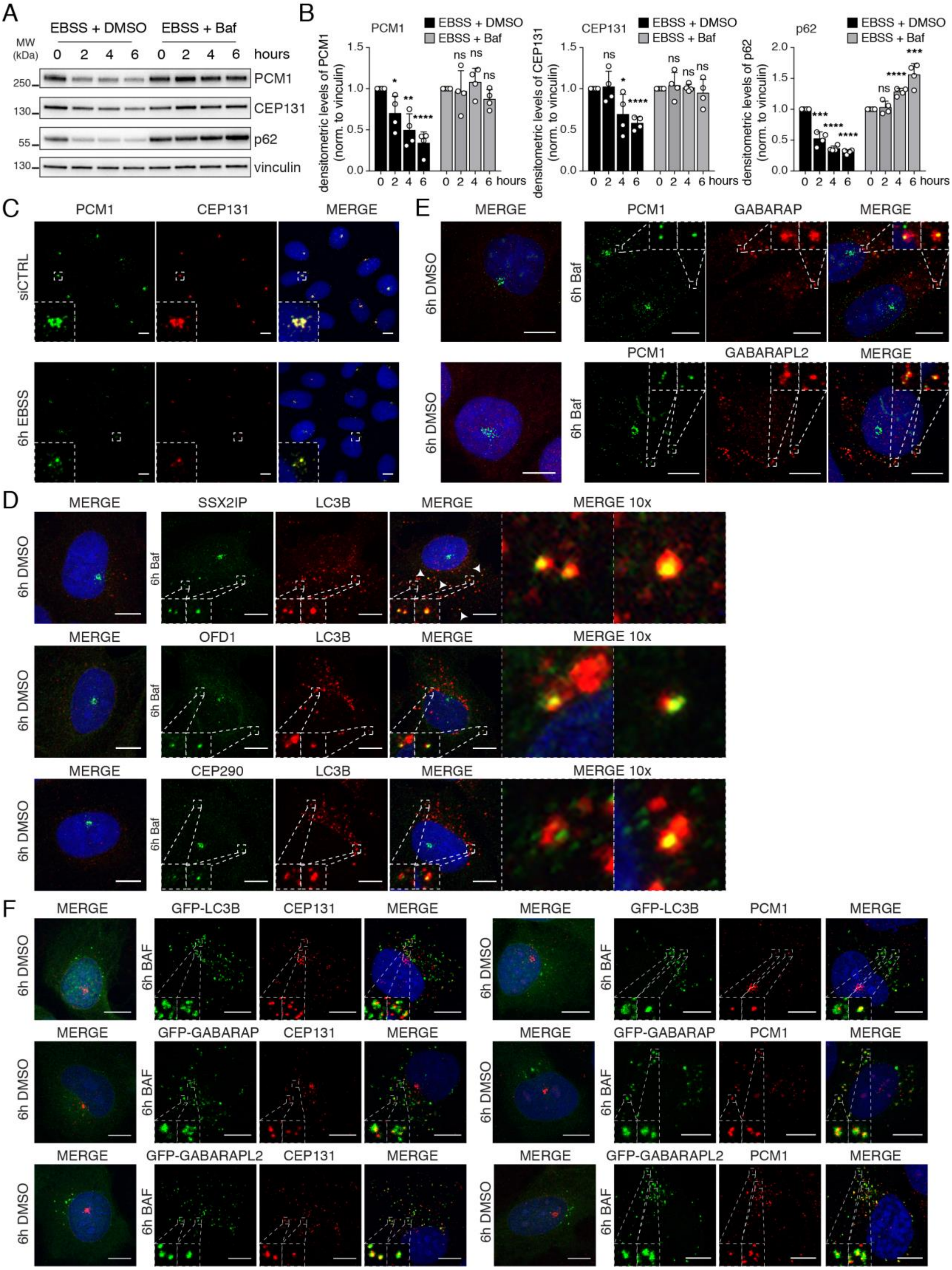

Continue on next page.

Figure S7b

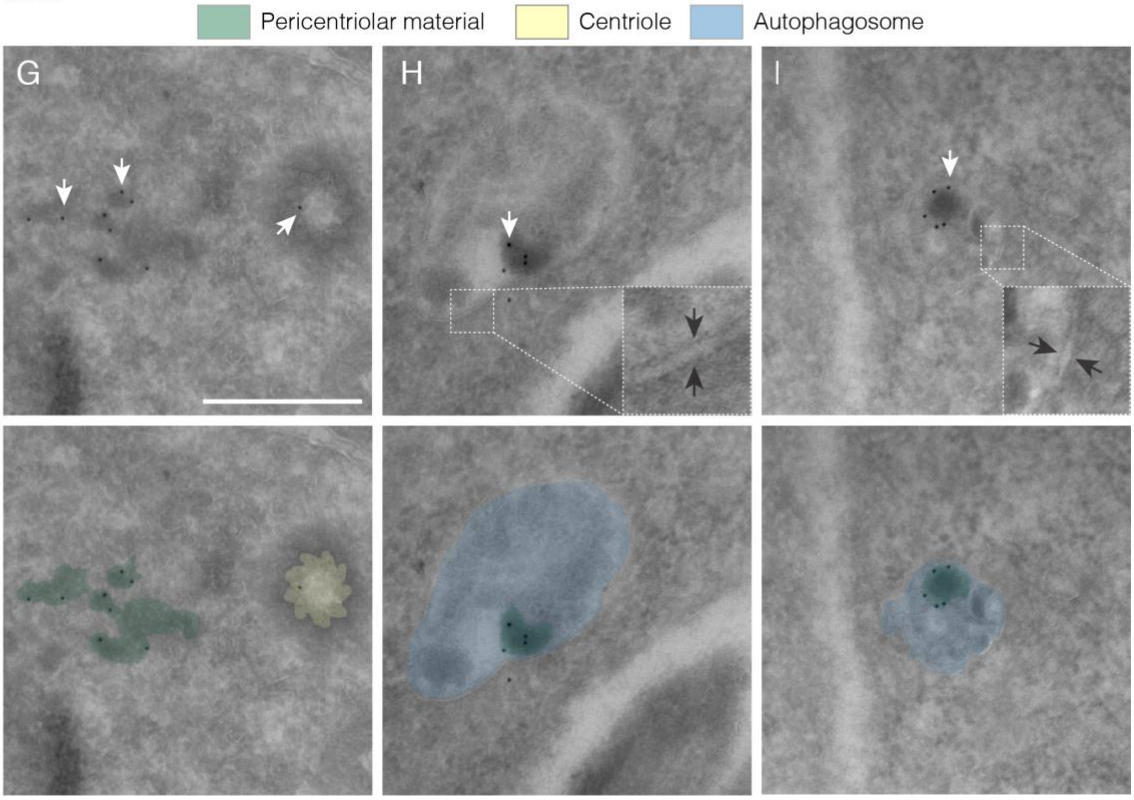

Supplementary Figure 7. Analysis of levels and localization of CS proteins upon autophagy induction. (A) Immunoblot of PCM1, CEP131 and p62 levels in U2OS cells incubated with EBSS + DMSO or EBSS + Baf for the indicated time points. **(B)** Densitometric levels of experiments represented in A. Columns represent mean ± SD, n = 3, * $P \leq 0.05$, ** $P \leq 0.01$, *** $P \leq 0.001$, **** $P \leq 0.0001$. Unpaired Student's *t*-test, two-tailed. **(C)** Co-staining of PCM1 and CEP131 in untreated cells or after 6 hours of starvation. **(D)** Co-localization of LC3B with SSX2IP, OFD1 or CEP290 in U2OS cells after 6 hours of Baf treatment. Arrows indicate points of colocalization. **(E)** U2OS cells showing co-localization of PCM1 with endogenous GABARAP or GABARAPL2 following 6 hours of treatment with Baf. **(F)** U2OS cells showing co-localization of PCM1 or CEP131 with GFP-tagged LC3B, GABARAP or GABARAPL2 following 6 hours of treatment with Baf. **(G-I)** U2OS cells were starved and treated with Baf for 4 hours. **(G)** Anti-PCM1 immunogold particles label pericentriolar structures (e.g., white arrows) and a centriole. **(H-I)** Two examples of presumptive autophagosomal structures (see double membranes indicated by black arrows in the inserts) also labelled by anti-PCM1 particles. The lower panels reproduce the panel row above with color codes indicating different organelles. Cells were exposed to Baf treatment. Scale bar, 500 nm. Scale bars, 10 µm, but for panels H-I. Source data are provided as a Source Data file.

Supplementary Figure 8

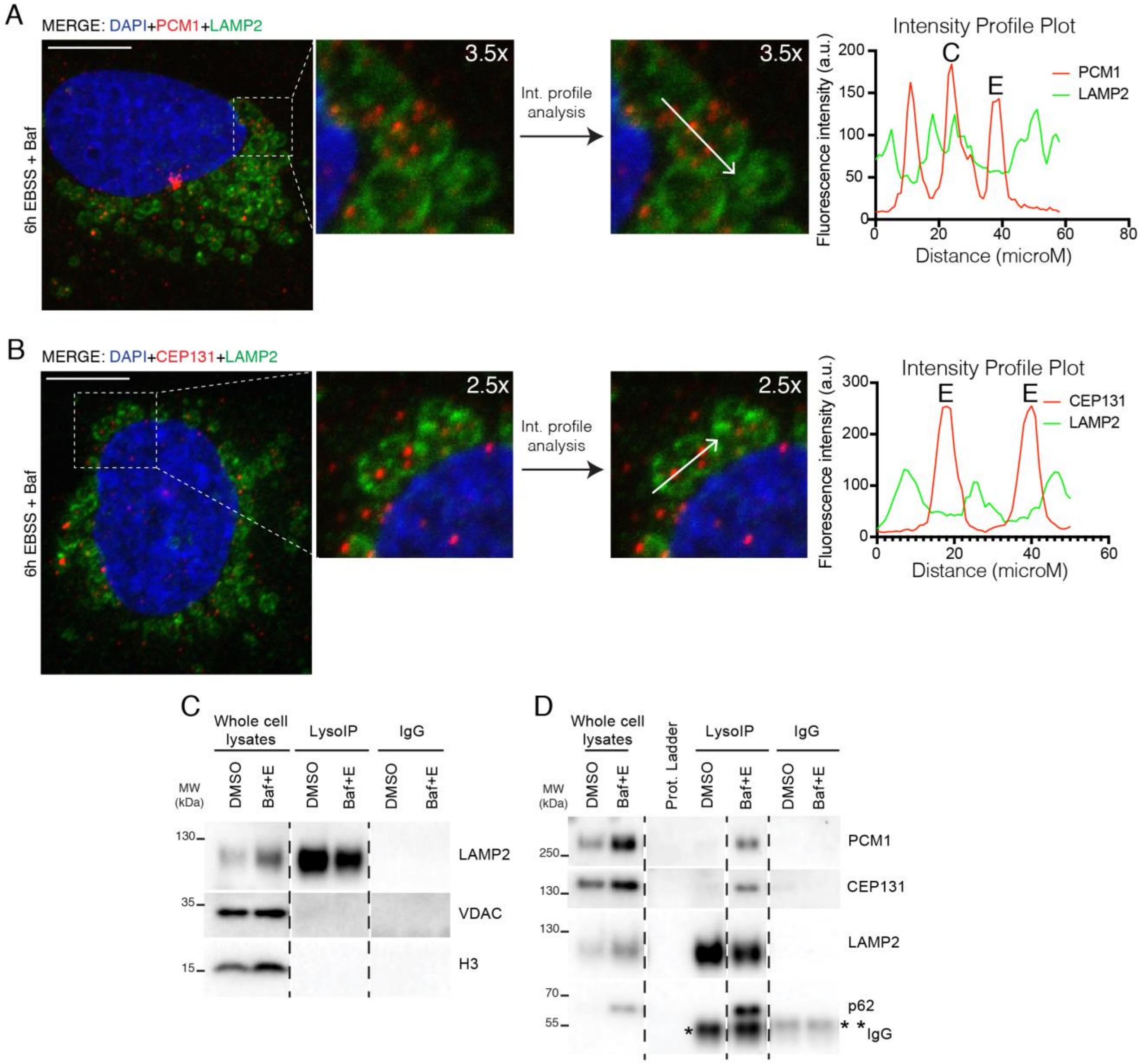

Supplementary Figure 8. PCM1 and CEP131 localise at the lysosome upon Baf treatment. (A) Co-localization of LAMP2 with endogenous PCM1 in U2OS cells treated for 4 hours with EBSS and Baf and stained for LAMP2, PCM1 and DAPI. On the right, the average of the Fluorescence Intensity profiles for the line in white is shown. C indicates PCM1 peaks of fluorescence correlating with LAMP2, while E indicates peaks falling between 2 LAMP2 peaks, most likely corresponding to the lysosomal membrane surrounding PCM1. **(B)** Co-localization of LAMP2 with endogenous CEP131 in U2OS cells treated as in J and stained for LAMP2, CEP131 and DAPI. Images and the corresponding Fluorescence intensity profiles are acquired/calculated and shown as in J. **(C)** Immunoblot of LAMP2 (lysosomal marker), VDAC (mitochondrial marker) and Histone H3 (nuclear marker) levels in immunopurified lysosomes obtained from U2OS cells treated with DMSO or EBSS and Baf for 4 hours. The vertical line represents a splice mark. The samples were obtained and processed in the same experiment and run/blot on the same gel/membrane. **(D)** Immunoblot of PCM1, CEP131, LAMP2 and p62 in the same immunopurified lysosomes shown in L. The vertical line represents a splice mark. The samples were obtained and processed in the same experiment and run/blot on the same gel/membrane. Scale bars, 10 µm.

Supplementary Figure 9

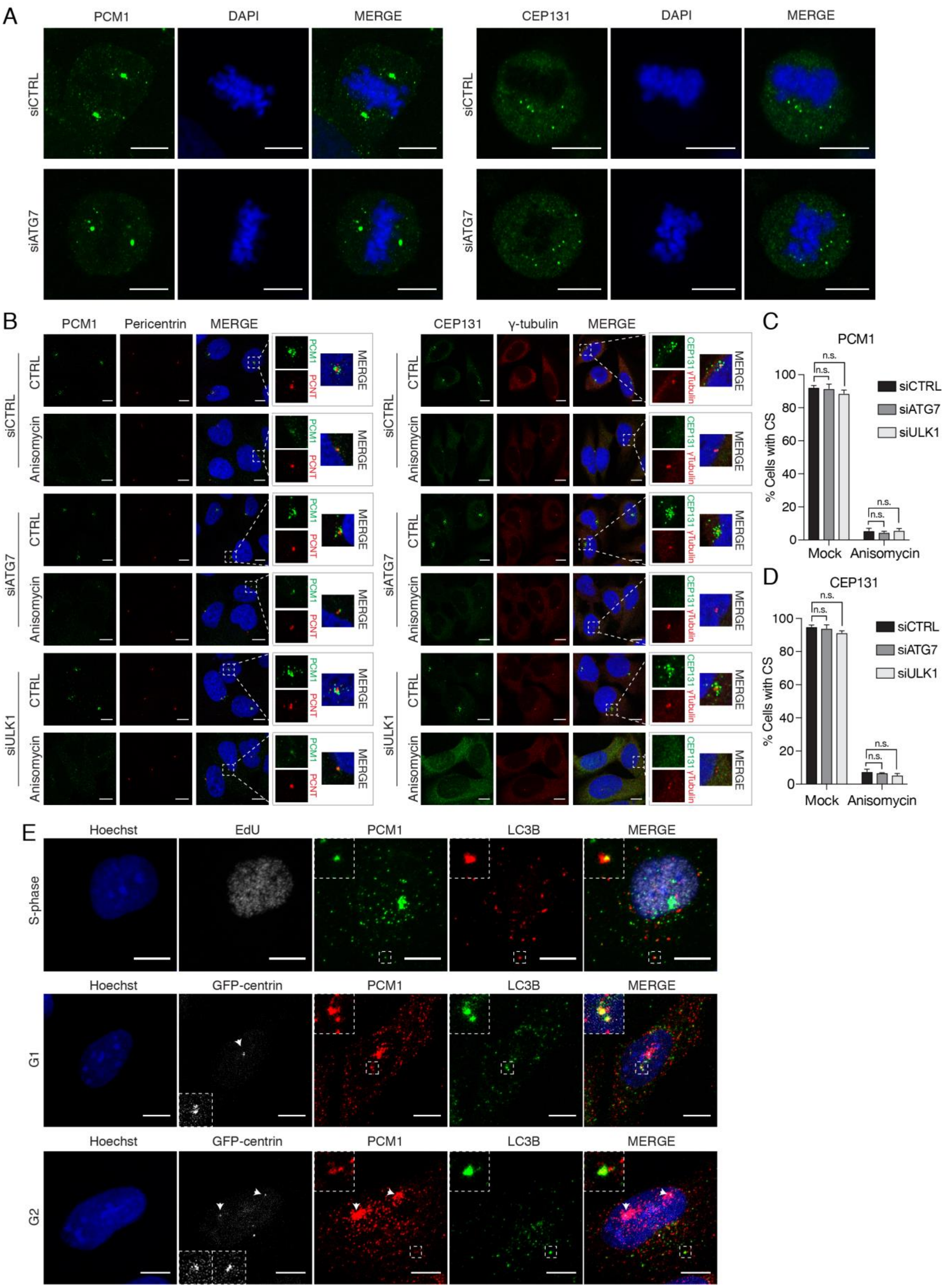

Supplementary Figure 9. CS dissociation during mitosis and upon stress stimuli is not affected by autophagy. (A) Representative images showing residual PCM1 and CEP131 satellites in mitotic U2OS cells treated with control or ATG7 siRNAs. **(B)** Anisomycin-induced centriolar satellite dissolution in U2OS cells treated with control, ULK1 or ATG7 siRNA. Cells were treated with 1 µg/ml anisomycin for 2 hours, fixed and stained for PCM1 and Pericentrin or CEP131 and γ-tubulin for evaluation of CS status. **(C-D)** Quantification of the percentage of cells displaying CS represented by PCM1 (C) or CEP131 (D), exemplified in B. Columns represent means ± S.D. n = 3 independent experiments. Two-way ANOVA with Dunnett's multiple comparisons test. (**E)** Representative images showing co-localization between LC3B and PCM1 in S, G1 and G2 phase cells. S-phase cells were identified by EdU staining. G1 and G2 cells were identified using U2OS cells stably expressing GFP-centrin by centriole status, G1 - a single centriole pair, G2 - separated duplicated centrosomes (late G2). Cells were treated for 2 hours with Baf prior to fixation to allow observation of a sufficient number of autophagosomes. Arrows indicate centrioles. Scale bars, 10 µm. Source data are provided as a Source Data file.

Supplementary Figure 10

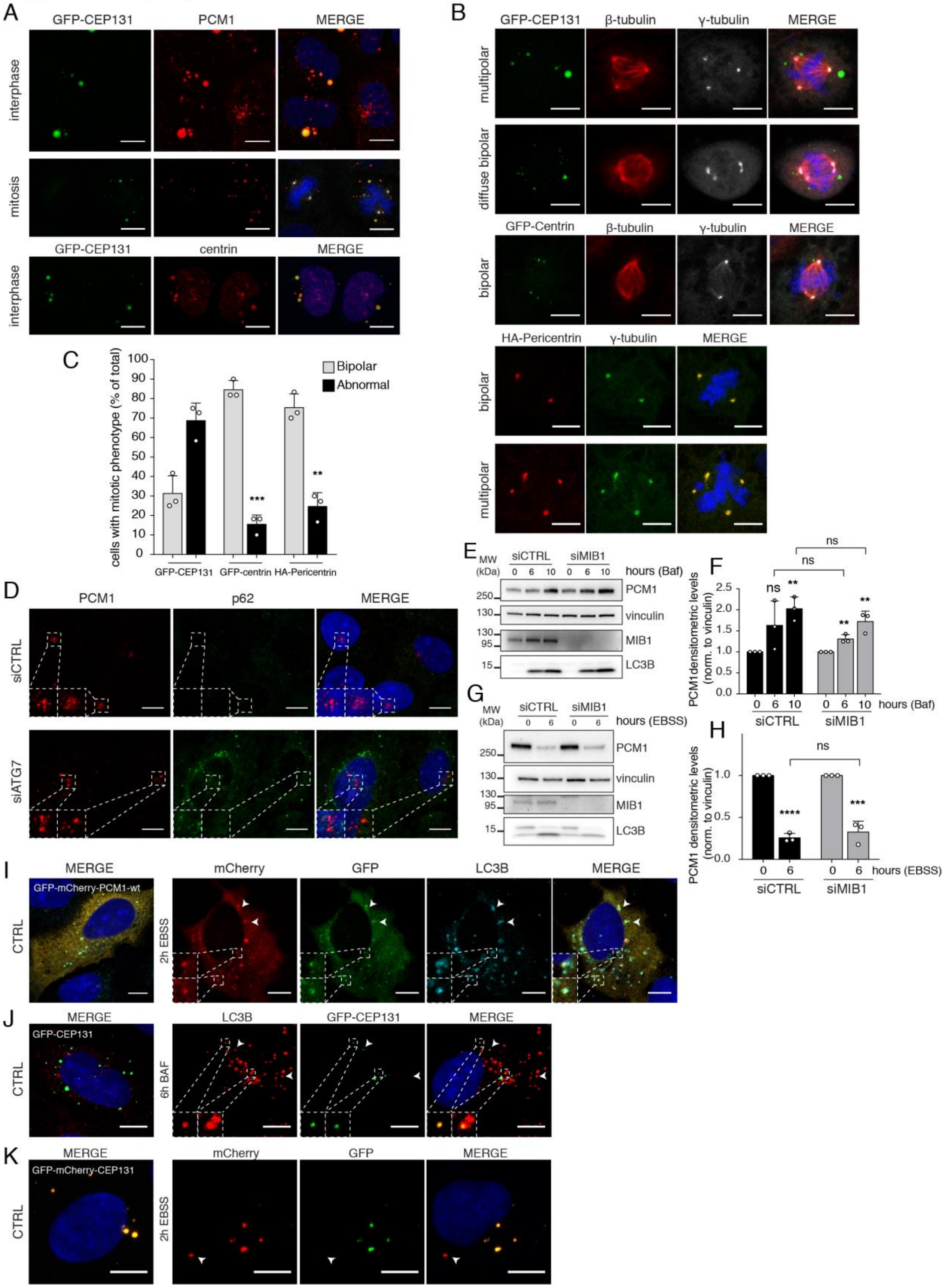

Supplementary Figure 10. Effect on mitosis of CEP131 over-expression, validation of the traffic-light-assay constructs and assesment of p62/MIB1 relevance to doryphagy. (A) GFP-CEP131 expressing cells evaluated for PCM1 and centrin co-localization. **(B)** Mitotic phenotypes in U2OS cells transiently expressing GFP-CEP131, GFP-centrin or HA-Pericentrin. Cells were stained for β-tubulin, γ-tubulin and Hoechst33342 (GFP-CEP131 and GFP-centrin) or HA, γ-tubulin and Hoechst33342 (HA-Pericentrin). **(C)** Quantification of phenotype distribution in B. Abnormal = multipolar, diffuse bipolar and monopolar mitoses combined. Columns represent the mean ± SD, n = 3 of ≥ 10 cells, ** $P \leq 0.01$, *** $P \leq 0.001$. Unpaired Student's *t*-test, two-tailed. **(D)** U2OS cells treated with control or ATG7 siRNA stained for PCM1, p62 and Hoechst33342. **(E)** Immunoblot of U2OS cells treated with control or MIB siRNA and subjected to Baf for the indicated intervals for evaluation of PCM1 accumulation. Vinculin is used as loading control. **(F)** Densitometric quantification of PCM1 levels in E. Columns represent the mean ± SD, n = 3, ns $P > 0.05$, ** $P \leq 0.01$. Unpaired Student's *t*-test, two-tailed. **(G)** Immunoblot of U2OS cells treated with control or MIB1 siRNA and incubated with EBSS as indicated for evaluation of PCM1 levels. Vinculin is used as loading control. **(H)** Densitometric quantification of PCM1 levels in G. Columns represent the mean ± SD, n = 3, ns, *** $P \leq 0.001$, **** $P \leq 0.0001$. Unpaired Student's *t*-test, two-tailed. **(I)** Traffic-light-assay of GFP-mCherry-PCM1 in U2OS cells following 2 hours of EBSS treatment. Cells are co-stained for LC3B for evaluation of autophagosomes-associated PCM1. Arrows indicate co-localization. **(J)** Co-localization between GFP-CEP131 and LC3B-stained autophagosomes after 6 hours of Baf treatment. Arrows indicate additional points of co-localization. **(K)** Representative image of GFP-mCherry-CEP131 after 2 hours of EBSS treatment showing formation of yellow and red foci, the latter indicating localization in lysosomes. Arrows indicate red foci. Scale bars, 10 µm. Source data are provided as a Source Data file.

Supplementary Figure 11

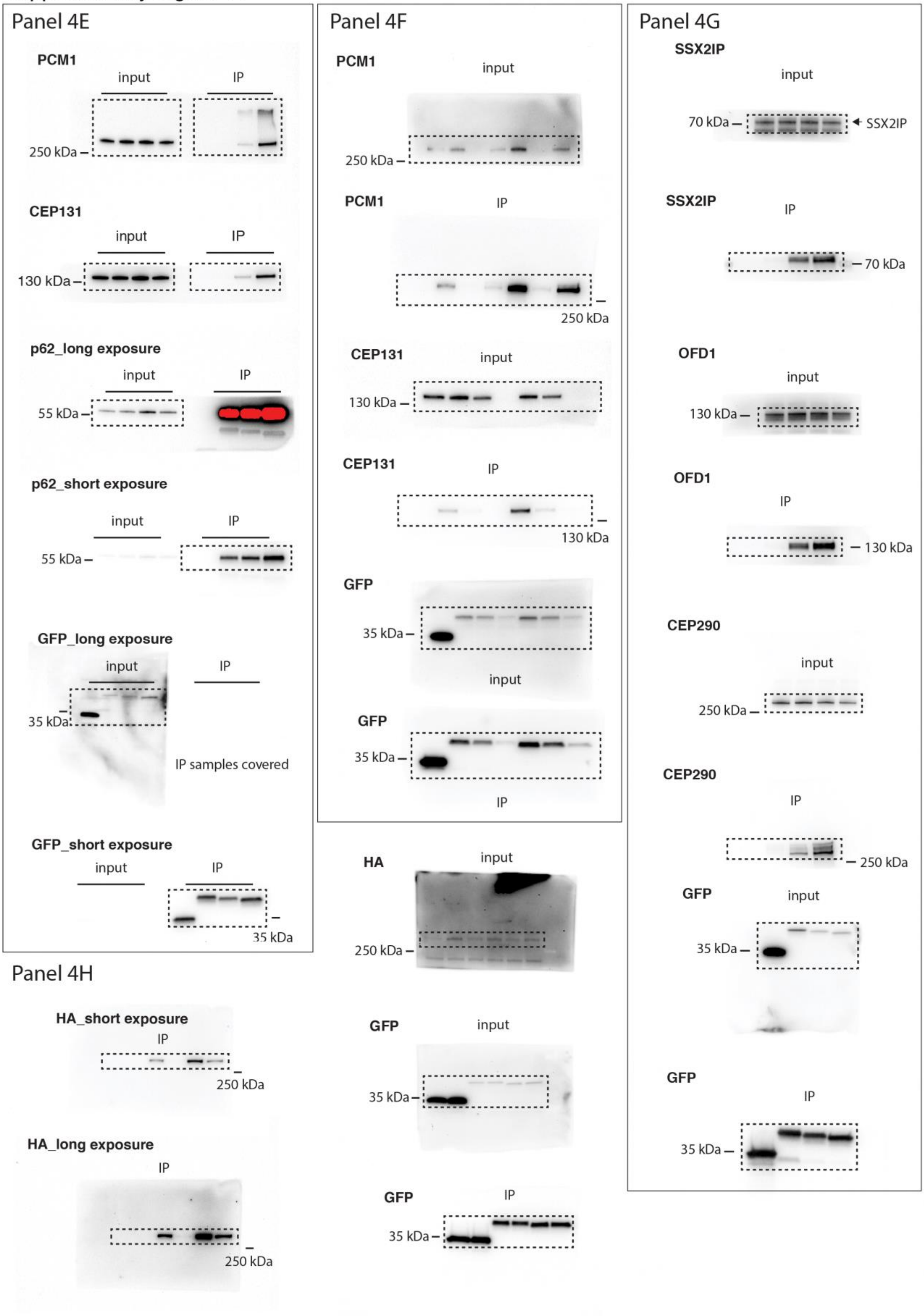

Continue on next page.

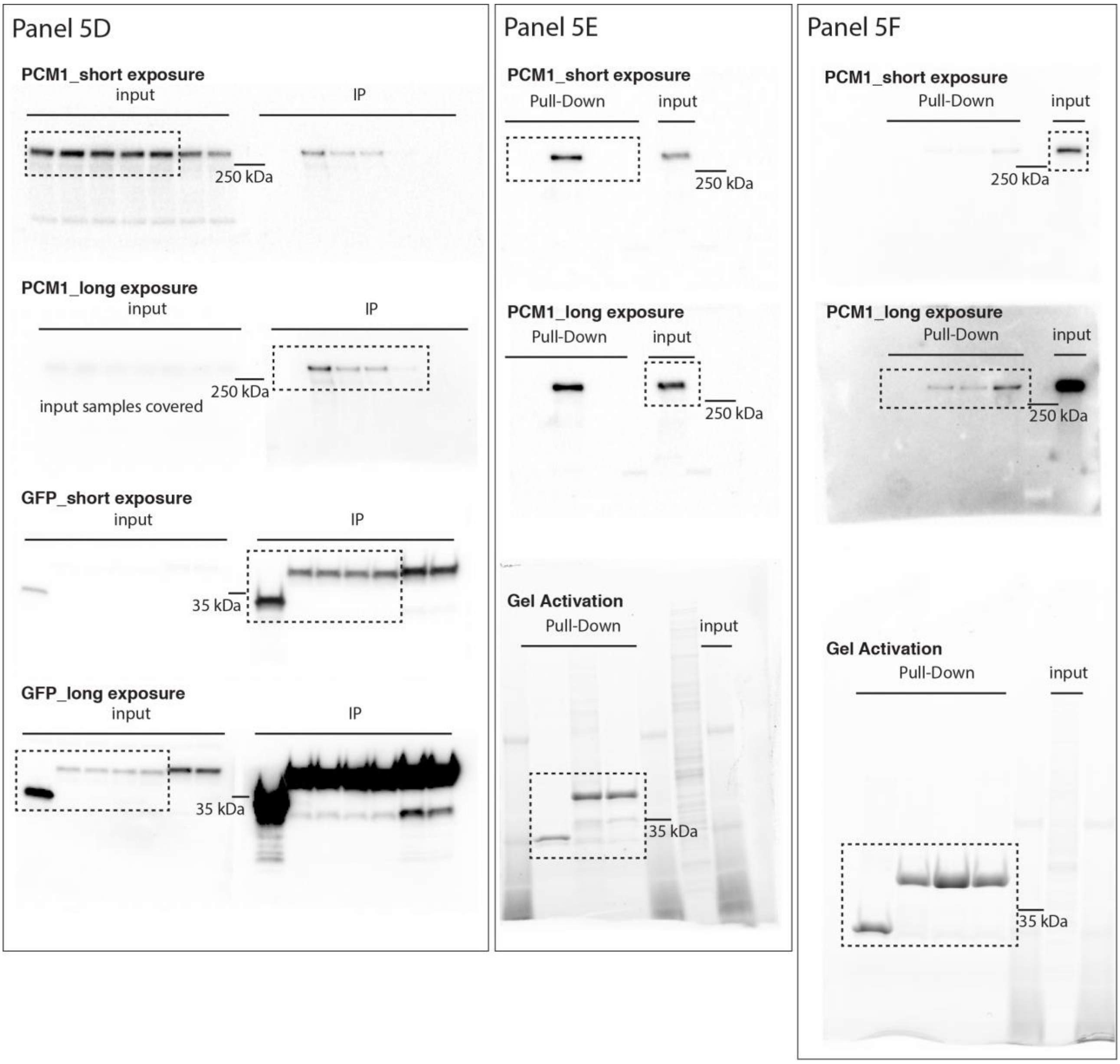

Continue on next page.

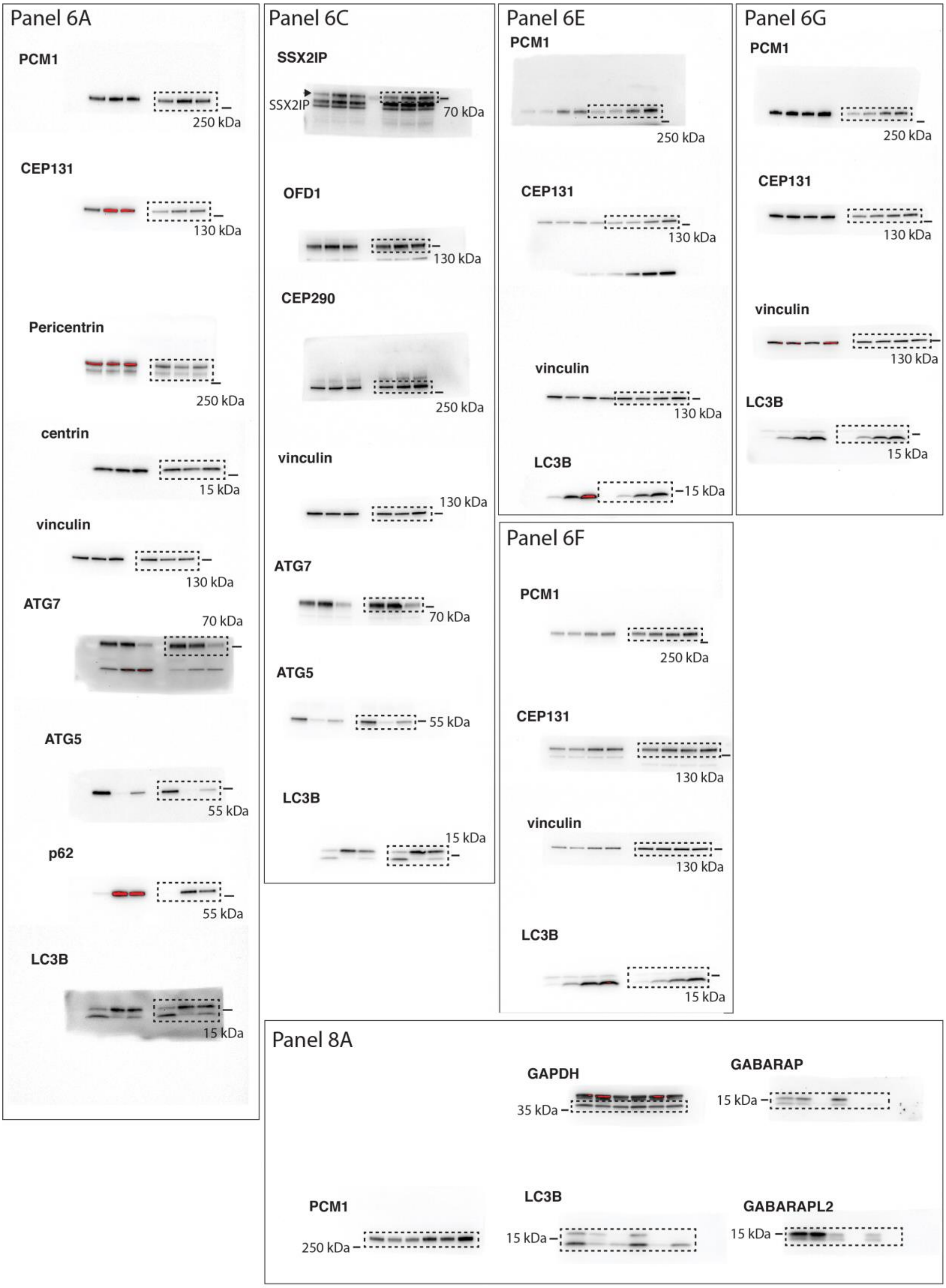

Continue on next page.

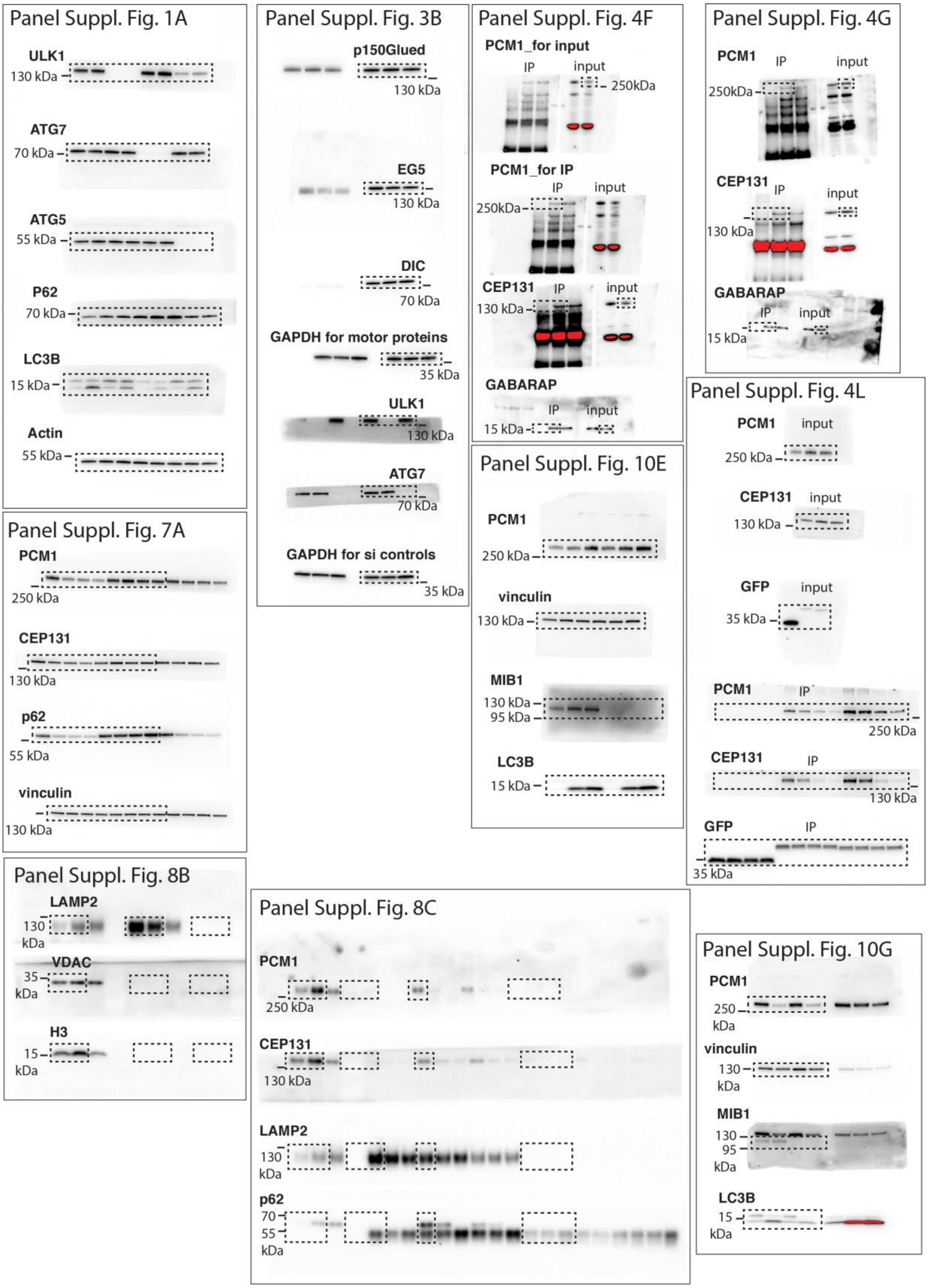

Supplementary Figure 11. Uncropped files of the western blots in the indicated figures.

Supplementary Table 1. Thermodynamic parameters of interactions between PCM1 LIR peptide and human ATG8-proteins.

	ΔH kcal mol^{-1}	ΔS cal mol^{-1} K^{-1}	-T*ΔS kcal mol^{-1}	ΔG kcal mol^{-1}	K_A *10^4 M^{-1}	K_D μM	N
**GABARAP**							
**WT**	-1.52±0.08	+18.7	-5.58	-7.09	15.7±1.4	6.4	0.94±0.04
**Y25H**	-1.00±0.04	+16.1	-4.80	-5.80	1.79±0.12	56	1.00*
**GABARAPL2**							
**WT**	+0.99±0.03	+28.4	-8.47	-7.45	31.1±2.6	3.2	0.98±0.02
**LC3B**							
**WT**	-0.89±0.02	+15.9	-4.74	-5.63	1.32±0.06	76	1.00*
**H27Y**	-1.28±0.04	+16.8	-5.01	-6.29	4.02±0.30	25	1.00*

* N was fixed to 1.00 upon fitting.
ΔH is the enthalpy change upon binding, ΔS is entropy change upon binding, T is absolute temperature, ΔG is change of the Gibbs energy upon binding, K_A is association and K_D is dissociation constant, N is the number of binding sites.

Supplementary Table 2. Plasmids obtained from other labs and used in the study without any modification.

Plasmid	Backbone	Owner/Reference
YFP-PCM1	pEYFP-C	S. Tooze (The Francis Crick Institute, London, UK)[3]
YFP-PCM1-3XA	pEYFP-C	S. Tooze (The Francis Crick Institute, London, UK)[3]
EGFP-CEP131	pEGFP-C	S. Bekker Jensen (University of Copenhagen, Copenhagen, Denmark)
GFP-3xFLAG	pLVX-TetOne-Puro	A. Lund (Biotech Research & Innovation Centre, University of Copenhagen, Copenhagen, Denmark)
GFP-LC3B	pLVX-TetOne-Puro	A. Lund (Biotech Research & Innovation Centre, University of Copenhagen, Copenhagen, Denmark)
GFP-GABARAP	pLVX-TetOne-Puro	A. Lund (Biotech Research & Innovation Centre, University of Copenhagen, Copenhagen, Denmark)
GFP-GABARAPL2	pLVX-TetOne-Puro	A. Lund (Biotech Research & Innovation Centre, University of Copenhagen, Copenhagen, Denmark)
HA-Pericentrin	pTB701	M. Takahashi (Teikyo Heisei University, Japan)[4]
GFP-centrin		M. Bornens (Institut Curie, France)[5]
pCMV-myc-ATG7	pCMV-Myc	M. H. Ögmundsdóttir (University of Iceland, Iceland)[6]
mCherry-LC3B	pLPCX	G.M. Fimia (National Institute for Infectious Diseases Lazzaro Spallanzani, Rome, Italy)
GST	pGEX-4T1	I. Dikic (Goethe University, Frankfurt, Germany)[7]
GST-LC3B	pGEX-4T1	I. Dikic (Goethe University, Frankfurt, Germany)[7]
GST-GABARAP	pGEX-4T1	I. Dikic (Goethe University, Frankfurt, Germany)[7]

Supplementary Table 3. List of plasmids generated in our lab, with relative method and primers used.

Plasmid	Mutation	Method	Primers
HA-PCM1	None	NEBuilder HiFi DNA assembly; PCM1 cds amplified from YFP-PCM1	Fw: 5'-CATGGAGGCCCCGGGGATCCGTATGGCCACAGGAGGAGGT-3'<br>Re: 5'-GTGTGATGGATATCTGCAGAATTCTCATATACTCTGGGCTCCCACC-3'
HA-PCM1-3xA	Mutated LIR domain: D1962A, D1963A, V1966A	NEBuilder HiFi DNA assembly; PCM1 cds amplified from YFP-PCM1-3XA	Same as HA-PCM1
pcDNA3-EGFP-mCherry-HA-PCM1	None	NEBuilder HiFi DNA assembly; EGFP-mCherry cassette inserted into the HA-PCM1	Fw: 5'-GACCCAAGCTTGGTACAGCGGTTCAGGATCAGGTTCAGGAATGG-3'<br>Re: 5'-ATCCGAGCTCGGTACCCGGTTGCCGACTTGTACAGCTCGTCCATGCCGC-3'

pcDNA3-EGFP-mCherry-HA-PCM1-3XA	mutated LIR domain D1962A, D1963A, V1966A	NEBuilder HiFi DNA assembly; EGFP-mCherry inserted into the HA-PCM1-3XA	Same as pcDNA3-EGFP-mCherry-HA-PCM1
pcDNA3-EGFP-mCherry-HA-CEP131	None	NEBuilder HiFi DNA assembly; EGFP-mCherry cassette inserted into EGFP-CEP131	Fw: 5'-CATGGAGGCCCCGGGaGGATCCAAAGGCACCCGGGCC-3'<br>Re: 5'-GTGTGATGGATATCTGCAGAATTCTCACTTGGTACTTGGCGTGG-3'
GFP-GABARAP-E8R,H9R	None	Site directed mutagenesis of GFP-GABARAP with Q5 DNA polymerase, NEB	Fw: 5'-CAAAGAAAGGCGTCCGTTCGAGAAGCGCCGCT-3'<br>Re: 5'-CGCTTCTCGAACGGACGCCTTTCTTTGTACACGAACTTCAT-3'
GFP-GABARAP-Y25H	Y25H	Site directed mutagenesis of GFP-GABARAP with Q5 DNA polymerase, NEB	Fw: 5'-CGAAAGAAACACCCGGACCGGGTGC-3'<br>Re: 5'-CCGGGTGTTTCTTTCGGATCTTCTCGCC-3'
GFP-GABARAP-E8R,H9R,Y25H	E8R, H9R, Y25H	Site directed mutagenesis of GFP-GABARAP-E8R,H9R with Q5 DNA polymerase, NEB	Same as for GFP-GABARAP-Y25H
siRNA resistant ATG7	Mutant siRNA binding site 995-CAGTAGATCTGAATCTAAAGCTGAT-1019	Site-directed mutagenesis of pCMV-myc-ATG7 with Q5 DNA polymerase, NEB	Re: 5'-CATCAGcTTtAGATTcAGATCtACTGATGACTCAGCTAACCTTTTAGGG-3'<br>Fw: 5'-AGTCATCAGTaGATCTgAATCTaAAgCTGATGTGTTGGAGATTGG-3'
GST-GABARAP-E8R,H9R,Y25H	E8R, H9R, Y25H	Site-directed mutagenesis with Q5 DNA polymerase, NEB.	Y25H-Fw: 5'-CGAAAGAAACACCCGGACCGGGTGC-3'<br>Y25H-Re: 5'-GGTGTTTCTTTCGGATCTTCTCGCC-3'<br><br>E8R,H9R-Fw: 5'-CAAAGAAAGGCGTCCGTTCGAGAAGCGCCGCT-3'<br>E8R,HR9-Re: 5'-AACGGACGCCTTTCTTTGTACACGAACTTGGATCCACGCGG-3'
GST-LC3B-R10E,R11H,H27Y	H27Y, R10E, R11H	Site-directed mutagenesis with Q5 DNA polymerase, NEB.	H27Y-Fw: 5'-AGAGCAGTATCCAACCAAAATCCCGGTG-3'<br>H27Y-Re: 5'-TTTGGTTGGATACTGCTCTCGAATAAGTCGGACA-3'<br><br>R10E,R11H-Fw: 5'-GAACACACCTTCGAACAAAGAGTAGAAGATGTCCG-3'<br>R10E,R11H-Re: 5'-CTTTGTTCGAAGGTGTGTTCCTGCTTGAAGGTCTTCTCCGAC-3'

Supplementary References

1. Rogov, V. V *et al.* Structural and functional analysis of the GABARAP interaction motif (GIM). *EMBO Rep.* e201643587 (2017). doi:10.15252/embr.201643587
2. Rogov, V. V *et al.* Phosphorylation of the mitochondrial autophagy receptor Nix enhances its interaction with LC3 proteins. *Sci. Rep.* **7**, 1131 (2017).
3. Joachim, J. *et al.* Centriolar Satellites Control GABARAP Ubiquitination and GABARAP-Mediated Autophagy. *Curr. Biol.* **27**, 2123-2136.e7 (2017).
4. Matsuo, K. *et al.* Kendrin is a novel substrate for separase involved in the licensing of centriole duplication. *Curr. Biol.* **22**, 915–21 (2012).
5. Piel, M., Meyer, P., Khodjakov, A., Rieder, C. L. & Bornens, M. The respective contributions of the mother and daughter centrioles to centrosome activity and behavior in vertebrate cells. *J. Cell Biol.* **149**, 317–30 (2000).
6. Ogmundsdottir, M. H. *et al.* A short isoform of ATG7 fails to lipidate LC3/GABARAP. *Sci. Rep.* **8**, 14391 (2018).
7. Grumati, P. *et al.* Full length RTN3 regulates turnover of tubular endoplasmic reticulum via selective autophagy. *Elife* **6**, e25555 (2017).
