# Supplementary material for: Selective autophagy maintains centrosome integrity and accurate mitosis by turnover of centriolar satellites
Source: Nat Commun. 2019 Sep 13;10:4176. doi: 10.1038/s41467-019-12094-9 (PMC6744468; doi:10.1038/s41467-019-12094-9)
Supplement: Supplementary file 15 — Reporting Summary [file 41467_2019_12094_MOESM15_ESM.pdf]

nature research

Corresponding author(s): Francesco Cecconi

Last updated by author(s): Jul 30, 2019

Reporting Summary

Nature Research wishes to improve the reproducibility of the work that we publish. This form provides structure for consistency and transparency in reporting. For further information on Nature Research policies, see Authors & Referees and the Editorial Policy Checklist.

Statistics

For all statistical analyses, confirm that the following items are present in the figure legend, table legend, main text, or Methods section.

n/a	Confirmed	
☐	☒	The exact sample size (n) for each experimental group/condition, given as a discrete number and unit of measurement
☐	☒	A statement on whether measurements were taken from distinct samples or whether the same sample was measured repeatedly
☐	☒	The statistical test(s) used AND whether they are one- or two-sided<br>*Only common tests should be described solely by name; describe more complex techniques in the Methods section.*
☐	☒	A description of all covariates tested
☒	☐	A description of any assumptions or corrections, such as tests of normality and adjustment for multiple comparisons
☐	☒	A full description of the statistical parameters including central tendency (e.g. means) or other basic estimates (e.g. regression coefficient) AND variation (e.g. standard deviation) or associated estimates of uncertainty (e.g. confidence intervals)
☐	☒	For null hypothesis testing, the test statistic (e.g. F, t, r) with confidence intervals, effect sizes, degrees of freedom and P value noted<br>*Give P values as exact values whenever suitable.*
☒	☐	For Bayesian analysis, information on the choice of priors and Markov chain Monte Carlo settings
☒	☐	For hierarchical and complex designs, identification of the appropriate level for tests and full reporting of outcomes
☒	☐	Estimates of effect sizes (e.g. Cohen's d, Pearson's r), indicating how they were calculated

Our web collection on statistics for biologists contains articles on many of the points above.

Software and code

Policy information about availability of computer code

Data collection	ImageLab, ZEISS LSM700 Acquisition Software<br>For Molecular modelling and MD simulations: Modeller V9.15, THESEUS, Gromacs V5.1, iLIR, MobiDB<br>No custom algorithms or software that are not already described in the published literature were used.
Data analysis	GraphPad Prism V7, Excel 2016, Image J 1.51n, Scan^R Analysis software (Olympus), Spotfire software (TIBCO)<br>For MS analysis: MaxQuant software V1.5.3.30, R (samr and ggplot package), AmiGo2 (geneontology.org)<br>ITC data: ITC-Origin 7.0 software<br>For Molecular modelling and MD simulations: Gromacs V5.1<br>No custom algorithms or software that are not already described in the published literature were used.<br>No custom algorithms or software that are not already described in the published literature were used.

For manuscripts utilizing custom algorithms or software that are central to the research but not yet described in published literature, software must be made available to editors/reviewers. We strongly encourage code deposition in a community repository (e.g. GitHub). See the Nature Research guidelines for submitting code & software for further information.

Data

Policy information about availability of data

All manuscripts must include a data availability statement. This statement should provide the following information, where applicable:

- Accession codes, unique identifiers, or web links for publicly available datasets
- A list of figures that have associated raw data
- A description of any restrictions on data availability

The mass spectrometry proteomics data have been deposited to the ProteomeXchange Consortium via the PRIDE partner repository with the dataset identifier PXD014829. All the scripts, input and output used for the simulation and modelling parts are available at E. Papaleo Group Github repository https://github.com/ELELAB/PCM1_LIR. The raw ITC data are available from the corresponding author upon reasonable request.

The source data underlying all Main and Supplementary Figures are provided as a Source Data file.

Field-specific reporting

Please select the one below that is the best fit for your research. If you are not sure, read the appropriate sections before making your selection.

☒ Life sciences ☐ Behavioural & social sciences ☐ Ecological, evolutionary & environmental sciences

For a reference copy of the document with all sections, see nature.com/documents/nr-reporting-summary-flat.pdf

Life sciences study design

All studies must disclose on these points even when the disclosure is negative.

Sample size	No sample-size calculation was performed. Our sample sizes were as a standard 3 independent experiments. For experiments where small differences were quantified, 4 independent experiments were conducted. The number of experiments are denoted in figure legends. These samples sizes were sufficient as the experiments were stable and reproducible.
Data exclusions	No data were excluded
Replication	Replication of the experiments validated our findings
Randomization	Randomization was applied where relevant. Fields and cells acquired by microscopy for subsequent analysis were randomly selected.
Blinding	The investigators were not blinded during data acquisition and analysis. The processes of treatments, sample collection and conduction of the experiments made it impossible to be blinded. We used cell lines in our experiments.

Reporting for specific materials, systems and methods

We require information from authors about some types of materials, experimental systems and methods used in many studies. Here, indicate whether each material, system or method listed is relevant to your study. If you are not sure if a list item applies to your research, read the appropriate section before selecting a response.

Materials & experimental systems

n/a	Involved in the study	
☐	☒	Antibodies
☐	☒	Eukaryotic cell lines
☒	☐	Palaeontology
☒	☐	Animals and other organisms
☒	☐	Human research participants
☒	☐	Clinical data

Methods

n/a	Involved in the study	
☒	☐	ChIP-seq
☒	☐	Flow cytometry
☒	☐	MRI-based neuroimaging

Antibodies

Antibodies used	Primary antibodies were as follows: ATG5 Cell Signaling Technology, 12994S, WB 1:1000), ATG7 (Cell Signaling Technology, 8558S, WB 1:1000), Actin (Novus Biologicals, NB600-501, WB 1:5000), -tubulin (Sigma-Aldrich, T4026, IF 1:200), centrin (a gift from I. Cheeseman51, Whitehead Institute for Biomedical, Research, Cambridge, USA, IF 1:1000), CEP63 (Proteintech, 16268-1-AP, IF 1:200), CEP131/AZI1 (Bethyl, A301-799 425A, WB 1:1000; Abcam, ab84864, IF 1:300; Abcam, ab99379, WB 1:1000, IF 1:500), CEP290 (Abcam, 800 ab84870, IF 1:200, WB 1:1000), CPAP (Proteintech, 11517-1-AP, IF 1:200), DIC 1/2 (Santa Cruz Biotechnology, sc-13524, WB 1:2000), EG5 (BD Transduction Laboratories, 611186, WB 1:2000), GABARAP (Abgent, AP1821a, WB 1:1000), GABARAP (Cell Signaling Technology, 13733S, WB 1:1000, IF 1:100), GABARAPL2 (Abcam, ab126607, WB 1:1000, IF 1:100), GAPDH (Merck-Millipore, CB1001, 1:20,000), GFP (Santa Cruz Biotechnology, sc-8334, WB 1:1000), -tubulin (Sigma-Aldrich, T3559, IF 1:200), HA (Sigma-Aldrich, H3663, WB 1:1000, IF 1:200), Histone H3 (Abcam, ab201456, WB 1:2000), LAMP1 (Abcam, ab24170, IP), LAMP2 (Abcam, ab25631, WB 1:2000; Developmental Studies Hybridoma Bank University of Iowa, H4B4, IF 1:100), LC3B (Cell Signaling Technology, 3868S, WB 1:1000, IF 1:200), LC3B (Cell Signaling Technology, 2775S, WB 1:1000), MIB1 807 (Novus Biologicals, NBP1-95846, WB 1:1000), NEDD1 (Santa Cruz Biotechnology, sc-100961, IF 1:200), NuMA (Abcam, ab36999, IF 1:200), OFD1 (Sigma-Aldrich, HPA031103, IF 1:200, WB 1:1000), p62/SQSTM1 (PM045, MBL International, WB 1:2000), p150Glued (BD Biosciences, 610473, WB 1:2000), PCM1 (Bethyl Laboratories, A301-150A, WB 1:1000, IF 1:500, EM 1:20), PCM1 (Santa Cruz Biotechnology, SC-398365, IF 1:200), Pericentrin (Abcam, ab4448, WB 1:1000, IF 1:200), SSX2IP (Sigma-Aldrich, HPA027306, IF 1:200, WB 1:1000), ULK1 (Cell Signaling Technology, 8054, WB 1:1000), VDAC (Cell Signaling Technology, 12454S, WB 1:1000), vinculin (Sigma-Aldrich, V4505, WB 1:2000).
Validation	All antibodies are validated by manufacturers. In addition, all autophagy markers were validate by us using proper positive controls, namely autophagy induction and Bafilomycin treatment (e.g. ATG8s lipidation/puncta localization upon autophagy

induction/Baf treatment, p62 level change upon autophagy induction/Baf treatment), as well as by knock-down. All centrosome and centriolar satellite markers were validate by localization experiments and/or knock-down.

Eukaryotic cell lines

Policy information about cell lines

Cell line source(s)	U2OS and HEK293 cells were obtained from ATCC.<br>MCF7 cells were obtained from ATCC and MCF7 GFP-tagged doxycycline-inducible cell lines were generated by standard lentiviral transduction procedure using the indicated constructs.<br>U2OS mRFP-tubulin H2B-GFP cells (mixed population) were provided by L. Lanzetti. (Serio et al. 2011)<br>MCF7-GFP-LC3 control were produced in Høyer-Hansen et al. 2007 and used for generation of ATG5 and ATG7 CRISPR/Cas9 KO cells by standard lentiviral transduction procedure using lentiCRISPR v2 constructs.
Authentication	The cell lines were not authenticated
Mycoplasma contamination	The cell lines were routinely tested for mycoplasma
Commonly misidentified lines<br>(See ICLAC register)	No ICLAC cell lines were used in the study.
